# Blended Interventions to Change Behavior in Patients With Chronic Somatic Disorders: Systematic Review

**DOI:** 10.2196/jmir.8108

**Published:** 2017-12-21

**Authors:** Corelien Kloek, Daniël Bossen, Dinny H de Bakker, Cindy Veenhof, Joost Dekker

**Affiliations:** ^1^ Tranzo Tilburg University Tilburg Netherlands; ^2^ Netherlands Institute for Health Services Research Utrecht Netherlands; ^3^ Research Group Innovation of Human Movement Care HU University of Applied Sciences Utrecht Netherlands; ^4^ Brain Center Rudolf Magnus Department of Rehabilitation, Physical Therapy Science & Sports University Medical Center Utrecht Utrecht Netherlands; ^5^ ACHIEVE Centre of Expertise Faculty of Health Amsterdam University of Applied Sciences Amsterdam Netherlands; ^6^ Coronel Institute of Occupational Health, Amsterdam Public Health Research Institute Academic Medical Center, University of Amsterdam Amsterdam Netherlands; ^7^ EMGO Institute VU University Medical Center Amsterdam Amsterdam Netherlands; ^8^ Department of Rehabilitation Medicine VU University Medical Center Amsterdam Amsterdam Netherlands; ^9^ Department of Psychiatry VU University Medical Center Amsterdam Amsterdam Netherlands

**Keywords:** telemedicine, chronic disease, behavior

## Abstract

**Background:**

Blended behavior change interventions combine therapeutic guidance with online care. This new way of delivering health care is supposed to stimulate patients with chronic somatic disorders in taking an active role in their disease management. However, knowledge about the effectiveness of blended behavior change interventions and how they should be composed is scattered.

**Objective:**

This comprehensive systematic review aimed to provide an overview of characteristics and effectiveness of blended behavior change interventions for patients with chronic somatic disorders.

**Methods:**

We searched for randomized controlled trials published from 2000 to April 2017 in PubMed, Embase, CINAHL, and Cochrane Central Register of Controlled Trials. Risk of bias was assessed using the Cochrane Collaboration tool. Study characteristics, intervention characteristics, and outcome data were extracted. Studies were sorted based on their comparison group. A best-evidence synthesis was conducted to summarize the effectiveness.

**Results:**

A total of 25 out of the 29 included studies were of high quality. Most studies (n=21; 72%) compared a blended intervention with no intervention. The majority of interventions focused on changing pain behavior (n=17; 59%), and the other interventions focused on lifestyle change (n=12; 41%). In addition, 26 studies (90%) focused on one type of behavior, whereas 3 studies (10%) focused on multiple behaviors. A total of 23 studies (79%) mentioned a theory as basis for the intervention. The therapeutic guidance in most studies (n=18; 62%) was non face-to-face by using email, phone, or videoconferencing, and in the other studies (partly), it was face-to-face (n=11; 38%). In 26 studies (90%), the online care was provided via a website, and in 3 studies (10%) via an app. In 22 studies (76%), the therapeutic guidance and online care were integrated instead of two separate aspects. A total of 26 outcome measures were included in the evidence synthesis comparing blended interventions with no intervention: for the coping strategy catastrophizing, we found strong evidence for a significant effect. In addition, 1 outcome measure was included in the evidence synthesis comparing blended interventions with face-to-face interventions, but no evidence for a significant effect was found. A total of 6 outcome measures were included in the evidence synthesis comparing blended interventions with online interventions, but no evidence for a significant effect was found.

**Conclusions:**

Blended behavior change interventions for patients with chronic somatic disorders show variety in the type of therapeutic guidance, the type of online care, and how these two delivery modes are integrated. The evidence of the effectiveness of blended interventions is inconsistent and nonsignificant for most outcome measures. Future research should focus on which type of blended intervention works for whom.

## Introduction

An important challenge of today’s health care is the management of patients with chronic somatic disorders. In addition, 1 out of 3 European adults deal with consequences of conditions such as heart failure, diabetes, asthma, or rheumatism [1]. Roughly, 50 million of them have even more than one chronic disorder (ie, multimorbidity) [2]. Patients’ behavior can influence the progression of their disorder and their perceived health, particularly when it concerns a lifestyle-related chronic disorder [3]. For those who need support in taking actions related to their lifestyle, a behavior change intervention can be helpful [4]. Examples are an education program for patients with rheumatoid arthritis [5] or an intervention for patients with chronic obstructive pulmonary disease (COPD) focused on physical activity, smoking, disease knowledge, and emotional wellbeing [6].

### Blended Interventions

An upcoming and new delivery mode for behavior change interventions is the use of Internet technologies, such as websites and apps. Although traditional behavior change interventions in primary care are restricted to face-to-face sessions, websites and apps are available at any time and place and can act as an extension of care provided by the professional. Online interventions without therapeutic guidance, however, struggle with disappointing adherence rates [7]. Therefore, it is recommended to combine online interventions with therapeutic guidance. The combination of online care and therapeutic guidance is called blended care, also known as technology supported care [7,8]. Bringing together the personal attention of a professional and the accessibility of an online tool is seen as a highly promising combination, which can stimulate patients to take an active role in their disease management [9]. The potential of integrating online care and technology within regular care for patients with chronic somatic disorders is also described in the recently developed eHealth Enhanced Chronic Care Model. The authors extended the original Chronic Care Model with eHealth tools to promote an informed and activated patient, to create productive interactions with the health care provider, and to increase patients’ self-management [10,11].

### Characteristics of Blended Interventions

Present blended interventions have in common that they consist of an online element complemented with therapeutic guidance; however, they show a wide variety in how both elements are delivered and combined. For example, the online part can be delivered via a website with solely information texts, but supplementary videos, games, and links can be used as well. In addition, the guidance by a therapist can be delivered in various ways, for example, by providing traditional face-to-face sessions, contact by email, or by videoconferencing [12]. One of the challenges in delivering blended care is the integration of online care and therapeutic guidance instead of two separate components [8]. When integrated properly, the website or app is not only supportive to the usual therapeutic guidance but is also a substantial element of the intervention as a whole [13].

Although blended care is seen as promising in terms of effectiveness and improving health care access, the actual usage in daily primary care practice is lagging behind [14]. More knowledge about the characteristics and the effectiveness of blended behavior change interventions may support the usage in daily health care practice. However, to our knowledge, a clear overview of blended behavior change interventions is missing in literature. We conducted a systematic literature review to investigate the characteristics and the effectiveness of blended behavior change interventions for patients with chronic somatic disorders. Chronic somatic disorders are defined as health conditions that are persistent or long-lasting [15]. Mental illnesses were excluded from this review. The first goal was to investigate the varieties of intervention characteristics of behavior change interventions in terms of type of online care, type of therapeutic guidance, the extent of online and therapeutic integration, and the theoretical basis of the intervention [16]. The second aim was to study the effectiveness of blended interventions for behavior change. The following questions were studied:

- Which types of blended behavior change interventions for patients with chronic somatic disorders are available in literature?
- What is the effectiveness in comparison with no intervention, face-to-face behavior change interventions, and online behavior change interventions without therapeutic guidance?

## Methods

### Search Strategy

A comprehensive literature search was conducted using PubMed, Embase, CINAHL, and Cochrane Central Register of Controlled Trials from January 2000 to April 2017. Studies published before 2000 were excluded because of the rapid developments within the field of eHealth. A combination of the following constructs was used: chronic somatic disorder, eHealth, behavior change intervention, and intervention study. Multimedia Appendix 1 shows the full range of keywords used for each construct.

Keywords were adapted to control vocabularies for different databases. Additionally, reference lists of included studies and other systematic reviews [13-18] were hand-searched for potentially relevant studies.

### Study Selection and Eligibility Criteria

First step of the study selection consisted of the screening of titles and abstracts of all retrieved studies on eligibility. This was performed by 2 researchers (CK and DB). Subsequently, full texts of all initially relevant studies were independently checked for inclusion by the same researchers. Disagreements about study inclusion were discussed until consensus was reached. Inclusion criteria are provided in Textbox 1. Studies on decision support systems or interventions using solely reminder messages as online component were excluded. Interventions in which the online component primarily consisted of health tracking technology or self-monitoring (eg, accelerometer or glucose meter) were also excluded, unless the tracking technology was integrated in a behavior change intervention with information and/or assignments.

### Data Extraction

Data were extracted from studies that met the inclusion criteria. These data comprised study characteristics (type of study, year of publication, type of control group, outcome measures, and timing of outcome assessment), study population (number of participants, age, sex, and type of chronic disorder), intervention characteristics (target behavior, described theoretical basis, duration of intervention, delivery mode and frequency of Internet-based element, delivery mode and frequency of therapeutic guidance, integration of online care, and therapeutic guidance), and type of control intervention. A modified version of the delivery coding schemes of Webb et al [16,17] was used for coding the Internet-based element: (1) assignments, (2) information, (3) enriched information environment (eg, supplementary content and links, videos, and games), (4) automated tailored feedback based on individual progress monitoring (eg, comparison with norms or goals, reinforcing messages, or coping messages), (5) automated follow-up messages (reminders, tips, and encouragement). Coded delivery modes for the therapeutic guidance were as follows: (1) option to request for advice (ask the expert, expert-led discussion board or chat sessions), (2) face-to-face contact, (3) email contact (scheduled), (4) phone calls, (5) short messaging service, (6) videoconferencing, and (7) discussion forum with peers. For the integration of therapeutic guidance and online care, we distinguished: (a) an integrated blended delivery mode for studies which mentioned that the therapeutic guidance was related to the content of the online care, for example, by discussing assignments or program progress, and (b) a nonintegrated blended delivery mode that was defined when the online care and the therapeutic guidance were described as two separate aspects or nothing was mentioned in the description of the therapeutic guidance about discussing or using a website or an app. Interventions in which the therapist only provided technical support and did not have access to online assignments and progress were also seen as nonintegrated.

Studies were sorted based on their type of control intervention: (1) no intervention, (2) face-to-face behavior change intervention, and (3) online behavior change intervention without therapeutic guidance.

All outcome measures were distracted and grouped into the following five constructs: (1) symptoms and signs, (2) limitations, (3) dealing with the chronic condition (cognitive and behavioral), (4) emotional outcomes, and (5) quality of life. Means and standard deviations for all outcome measurements (pre- and postvalues) were extracted. A *P* value of <.05 was considered a significant indication for effectiveness.

### Quality Assessment

All articles were independently assessed on methodological quality by 2 researchers (CK and DB). For this assessment, the risk of bias criteria list of the Cochrane collaboration was used [18]. A total of 10 dimensions were assessed, namely, random sequence generation (selection bias), allocation concealment (selection bias), blinding of outcome assessor (detection bias), incomplete outcome data (attrition bias), selective reporting of results (reporting bias), group similarity at baseline (selection bias), cointerventions (performance bias), compliance (performance bias), intention-to-treat analysis, and timing of outcome assessments (detection bias). The criteria of blinding of participants and personnel (performance bias) were not used, as blinding is not possible in the types of intervention investigated in this review. Each study was rated as low risk, high risk, or unclear when there were no data to assess this criterion. Dimensions scored as low risk received 1 point. Dimensions scored as high risk or unclear received 0 points.

**Inclusion criteria for this study.**

- randomized controlled trial published in the English language
- the patient sample comprised adults (≥18 years) with chronic somatic disorders
- the study included an intervention aimed to change one or more of the following behaviors: physical activity, dietary intake, pain coping, and time spent in sedentary activity
- the intervention consisted of a combination of online care provided through a website, app, or automatic email and contains at least two episodes of contact with a health care professional (either face-to-face, personal emails, telephone, or videoconference)
- the blended intervention was compared with waiting list or usual care, a face-to-face intervention, or an online intervention

**Table 1:** Best-evidence synthesis.

Level of evidence	Description
Strong evidence	Consistent findings in multiple (≥3) high-quality RCTs^a^
Moderate evidence	Consistent findings in at least one high-quality study and at least one low-quality study, or consistent findings in multiple low-quality studies
Inconsistent evidence	Inconsistent findings in multiple studies
Insufficient evidence	Only one or two studies available

^a^RCTs: randomized controlled trials.

Points were counted and summarized as a risk of bias score (range 0-10, where 10 indicates low risk of bias for all 10 dimensions). Studies with a score of ≥6 were judged as high methodological quality. Interobserver agreement was expressed as the percentage of agreement on bias dimensions between CK and DB.

### Data Analysis

A best-evidence synthesis was conducted to summarize the effectiveness of blended behavior change interventions, using the same method used by Proper et al [19]. For this synthesis, the number of studies, methodological quality, and consistency of findings were all taken into account. A distinction was made for each of the 3 types of control conditions. Outcome measurements that were measured 3 times or more were sorted on level of evidence: strong evidence, moderate evidence, and inconsistent evidence (Table 1). When there were at least three high methodological quality studies, studies with low quality were disregarded from the evidence synthesis. When at least 75% of the studies showed results in the same direction, results were considered consistent. In case of 3-arm studies, all eligible between-group comparisons were included and treated as different studies.

## Results

### Search Results and Study Characteristics

**Figure 1:**
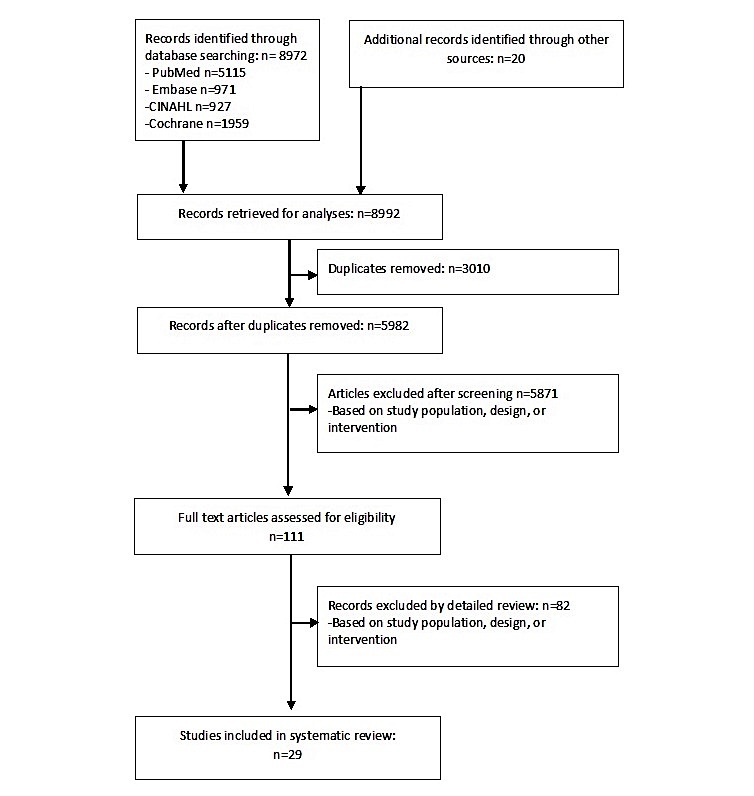
Flowchart of selection procedure.

The initial literature search resulted in 8992 articles. After deleting duplicates, 6192 unique articles were screened on title and abstract. A total of 111 selected articles were studied on full text, whereof 29 articles met the inclusion criteria. An overview of the selection procedure is shown in Figure 1.

### Characteristics of Selected Studies

An overview of study characteristics is shown in Multimedia Appendix 2. Sample size ranged from 45 to 463 participants. A total of 17 interventions were targeted on changing pain thinking and pain behavior related to chronic pain [20,21], irritable bowel syndrome [22,23], chronic tinnitus [24], diabetes mellitus [25], multiple sclerosis [26], rheumatoid arthritis [27], fibromyalgia [28], psoriasis [29], and cancer [30]. Furthermore, 12 studies were targeted on changing lifestyle behavior (ie, physical activity, nutrition, and sedentary behavior) for patients with obesity [31,32], diabetes mellitus [33,34], chronic obstructive pulmonary disease [34], multiple sclerosis [35,36], and rheumatoid arthritis [37]. Moreover, 1 study was targeted on asthma self-management skills [38]. Out of all 29 included randomized controlled trials, 21 studies had 2 study arms, 5 studies had 3 study arms, and 3 studies used a 4-arm design. Divided per control group, 21 studies compared the blended intervention with no intervention, 5 studies made a comparison with a face-to-face intervention, and 10 studies compared a blended intervention with an online self-guided intervention. The number of outcome measures per study ranged from 1 to 21.

### Methodological Quality

Ten different sources of bias were rated to assess the methodological quality of the studies (Multimedia Appendix 3). There was 87% agreement between the reviewers. After discussion, consensus was reached and no third reviewer had to be consulted.

In total, 25 studies were rated as high quality [20,21,23-35,37,39,41-49] and 4 studies as low quality [22,36,38,40]. The most frequent sources of bias were not reporting blinding of the outcome assessor (90% of studies) and information about patients’ use of cointerventions (93% of studies).

### Characteristics of Blended Behavior Change Interventions

An overview of intervention characteristics is shown in Multimedia Appendix 2. The length of the interventions ranged from 5 weeks to 12 months. Most interventions focused on one target behavior [20-30,32,34-46,48], and 3 interventions were focused on multiple behaviors (ie, nutrition and physical activity) [31,33,47]. A total of 23 studies mentioned a theory as basis for the intervention, most frequently the principles of cognitive behavior therapy [20-22,24,26-30,32,43,44], social cognitive theory [31,36,45], and acceptance and commitment therapy [23,42,48]. In contrast, 6 studies did not mention any theory [34,35,37-39,47]. In 11 studies, the therapeutic guidance was delivered through face-to-face contact[21,30-33,37-39], mostly in combination with email or phone communication [27,29,30,32-34,37-39]. In 18 studies, the therapeutic guidance was non face-to-face [20,22-26,28,34,38,40-48]. In 12 studies, patients had the option to request for advice at a random moment [23-25,27,30,32,34,38,39,45-47]. Frequency of therapeutic guidance varied from weekly contact to bimonthly. A total of 22 studies delivered online care through a website, and the other 3 studies via an app [31,34,47]. Furthermore, 21 interventions were enriched with videos, links, games, automated tailored feedback r automated reminder messages, and in 8 studies, the online care consisted solely of assignments and information [22-24,29,37,39,40,45]. 

In 7 studies, nothing was mentioned about the use of the website or app during the therapeutic guidance, and therefore, they were classified as nonintegrated [21,24,29-31,37,47]. In all other interventions, the online care and the therapeutic guidance were described to be integrated. For example, in the study of De Boer et al [20], the psychologist emailed personal feedback on homework assignments.

In the study of Buhrman et al [43], the therapist tailored the online care by selecting treatment modules that were in line with the individual needs of the patient.

### Effectiveness of Blended Care Versus no Intervention

Multimedia Appendix 4 demonstrates 21 studies that compared a blended behavior intervention with no intervention. A complete overview with levels of evidence is given in Table 2. Within the construct of symptoms and signs, strong evidence for a nonsignificant effect was seen for pain reduction [27,28,30,40-44,48], fatigue reduction [27-30], and body weight reduction [32,39]. Within the construct of limitations, inconsistent evidence was found for disability improvement [23,43,44,48]. With regard to the construct dealing with the chronic condition: cognitive measures, strong evidence for a significant effect was found for reducing catastrophizing thoughts [22,28,40-43,48]. Inconsistent evidence was found for improving acceptance of the chronic condition [24,44], reducing fear of movement [28,44], improving pain self-efficacy [28,44], and the coping strategy praying or hoping [40-43]. 

**Table 2:** Effectiveness of blended behavior change interventions compared with no intervention, face-to-face behavior change intervention, and online behavior change intervention.

Control conditions and constructs			Outcome construct
**No intervention**			
	**Symptoms and signs**		
		Pain	Strong evidence for a nonsignificant effect
		Fatigue	Strong evidence for a nonsignificant effect
		Body weight	Strong evidence for a nonsignificant effect
	**Limitations**		
		Disability	Inconsistent evidence
	**Dealing with the chronic condition: cognitive measures**		
		Coping strategy: catastrophizing	Strong evidence for a significant effect
		Acceptance	Inconsistent evidence
		Coping strategy: praying or hoping	Inconsistent evidence
		Fear of movement	Inconsistent evidence
		Pain self-efficacy	Inconsistent evidence
		Coping strategy: diverting attention	Strong evidence for a nonsignificant effect
		Coping strategy: reinterpret pain sensation	Strong evidence for a nonsignificant effect
		Coping strategy: coping self-statements	Strong evidence for a nonsignificant effect
		Coping strategy: ignore pain sensations	Strong evidence for a nonsignificant effect
		Perceived life control	Strong evidence for a nonsignificant effect
		Perception of support received from others	Strong evidence for a nonsignificant effect
		Perception of received punishing responses	Strong evidence for a nonsignificant effect
		Perception of received solicitous responses	Strong evidence for a nonsignificant effect
		Perception of received distracting responses	Strong evidence for a nonsignificant effect
	**Dealing with the chronic condition: behavior measures**		
		Coping strategy: increase activity level	Strong evidence for a nonsignificant effect
		Pain interference with daily activities	Strong evidence for a nonsignificant effect
	**Emotional outcomes**		
		Anxiety	Inconsistent evidence
		Depression	Inconsistent evidence
		Affective distress	Inconsistent evidence
	**Quality of life**		
		Generic quality of life	Inconsistent evidence
		Health-related quality of life: emotional role impairment	Inconsistent evidence
		Health-related quality of life: emotional role impairment	Inconsistent evidence
**Face-to-face behavior change intervention**			
	**Limitations**		
		Physical activity	Inconsistent evidence
**Online behavior change intervention**			
	**Symptoms and signs**		
		Pain	Inconsistent evidence
		Body mass index	Inconsistent evidence
		Body weight	Strong evidence for a nonsignificant effect
	**Limitations**		
		Physical activity	Inconsistent evidence
	**Emotional outcomes**		
		Anxiety	Strong evidence for a nonsignificant effect
		Depression	Inconsistent evidence

Strong evidence for a nonsignificant effect was found for the coping strategies diverting attention, reinterpret pain sensations, coping self-statements and ignorance of pain sensations, perceived life control, perception of support received from others, perception of received punishing responses, perception of received solicitous responses, and perception of received distracting responses [40-43].

Within the construct dealing with the chronic condition: behavioral measures, strong evidence for a nonsignificant effect was found for pain interference with daily activities [28,40-43,48] and strong evidence for a nonsignificant effect was found for the coping strategy increasing activity level [40-43]. Within the construct emotional outcomes, inconsistent evidence was found for reducing anxiety [22,24,26-29,40-44,48], depression [24,26-30,40-44,48], and affective distress [40-43]. Inconsistent evidence was also found for the improvement of generic quality of life [41-43] and emotional and physical health-related quality of life [27-29,34].

### Effectiveness of Blended Care Versus Face-to-Face

Multimedia Appendix 5 demonstrates 5 studies that compared a blended behavior intervention with a face-to-face behavior change intervention. A complete overview with levels of evidence is given in Table 2. Within the construct limitations, inconsistent evidence was found for increasing levels of physical activity [31,34]. All other outcome measures were measured less than 3 times, indicating insufficient evidence.

### Effectiveness of Blended Care Versus Online Care

Multimedia Appendix 6 shows 10 studies that compared a blended behavior intervention with an online behavior change intervention. A complete overview with levels of evidence is given in Table 2. Within the construct symptoms and signs, inconsistent evidence was found for reduction of pain [44,48] and body mass index [31,33]. Strong evidence for a nonsignificant effect was found for body weight reduction [31-33]. Within the construct limitations, strong evidence for a nonsignificant effect was found for improving physical activity levels [31,33,37,45,46]. Within the construct emotional outcomes, strong evidence for a nonsignificant effect was found for reducing anxiety [44,48] and depression [25,44,46,48].

## Discussion

### Principal Findings

This review provides an overview of the intervention characteristics of a new and promising field within health care for patients with chronic somatic disorders. The characteristics of the included blended behavior change interventions showed a wide heterogeneity. For example, length of interventions ranged from 5 weeks to 12 months. A previous systematic review that studied factors related to online adherence showed that shorter interventions are related to higher usage rates [50]. On the other hand, it is also known that long-term maintenance of behavior change is challenging [51] and that an extension of the intervention with follow-up booster sessions improves the overall effectiveness of face-to-face interventions [52]. The majority of interventions focused on one type of behavior. As many people have multiple unhealthy behaviors linked to risk factors for different chronic diseases, studies should focus on changing multiple behaviors [4]. Such holistic programs have a great potential for targeting complete health profiles and stimulating patients to take an active role in their health management.

The theoretical basis of the intervention content was most frequently based on the principles of cognitive behavior therapy. The aim of the cognitive behavior therapy is to change individuals’ unhelpful thoughts, beliefs, and behaviors [53]. In less than half of the studies, the therapeutic guidance was delivered face-to-face, whereas in the other studies, it was delivered completely at distance. Future research is needed to investigate whether face-to-face contact, guidance at distance, or a combination of multiple delivery modes are more or less effective for the overall effectiveness of a blended intervention. The review of Webb et al [16] showed that an “ask the expert” facility is related to higher effectiveness. This additional option was used in 12 out of 29 studies. Furthermore, it is known that the use of an enriched information environment is related to higher effectiveness [16]. Such supplementary content, such as videos and links to informative websites, was used in most interventions. In summary, we can conclude that a wide diversity was seen in the characteristics or ingredients of blended interventions. Given the considerable heterogeneity in the interventions, it was difficult to isolate subtypes of blended interventions for patients with chronic somatic disorders. Future research should focus on which type of blended intervention works for whom, for example, by using subgroup analyses and comparing different types of blended care.

Almost all included studies described that the therapeutic guidance and the online care were integrated with each other. Examples of integration of therapeutic guidance and online care were the provision of therapeutic feedback on online assignments or tailoring of the online intervention by the therapist. This high number of integrated blended interventions surprised us, as in literature, the interconnection of the therapeutic and the Web-based part is described as one of the biggest challenges of blended care [8,54]. When Web-based apps are integrated within health care, online care is often used as an additional component to usual care, instead of being a substantial element of the intervention as a whole [8]. Although the interventions were described as interconnected, analyses of user experiences are needed to draw conclusions about actual experienced integration.

A wide range of outcome measures were included in our evidence synthesis comparing blended interventions with no interventions or online blended interventions without therapeutic guidance. For some outcome measures, we found inconsistent evidence, and for other outcome measures, we found strong evidence for a nonsignificant effect. The lack of evidence for blended interventions, even when comparing with no intervention, is surprising. Although blended care is described as best of both worlds [8], results of this systematic review do not support this expectation. Before broad-scale implementation of blended behavior change interventions in daily practice, further investigation of how blended interventions should be composed is needed.

A minority of studies compared blended interventions with face-to-face interventions. The evidence synthesis of this comparison showed inconsistent evidence for improvement in physical activity. Particularly, for the comparison of blended behavior change interventions with face-to-face interventions, it would be interesting to investigate cost-effectiveness, long-term effectiveness, and patient satisfaction. The potential added value of blended care above face-to-face care may be found in these outcome measures instead of outcome measures related to symptoms and signs, limitations, behavior, emotions, and quality of life. To illustrate, if face-to-face sessions are substituted by online care, blended interventions may be cheaper than usual care [55]. Another advantage of blended interventions over face-to-face care is the possibility to overcome geographical barriers, as therapeutic guidance in these interventions can be served by a computer or mobile phone.

### Limitations

A methodological limitation of our evidence synthesis is the use of multiple outcome measures and multiple comparisons. This multiplicity may result in an increased risk of false-positive statistically significant indications of the effectiveness of blended behavior change interventions [56]. Moreover, 4 studies were conducted by the same research group [40-43]. These 4 studies investigated interventions targeted on the same behavior and generally used the same measurement instruments. The predominance of these 4 studies within the evidence synthesis may also lead to false-positive statistically significant indications of the effectiveness of blended behavior change interventions.

### Implications for Future Research

This review investigated a huge heterogeneity in how blended interventions were composed. For future research, we suggest investigating the effectiveness of different intervention components such as intervention duration, type of face-to-face guidance, and type of online care. Studies included in this review provided the same intervention, with the same amount of ingredients to the entire group of included patients. However, with respect to individual differences, it is presumed that different patients benefit from different blended interventions. For example, considering the ratio between online care and therapeutic guidance, one patient may benefit from more online support, whereas others need more therapeutic guidance. To determine the most optimal ratio in the treatment of patients with depression, the Fit for blended care instrument was recently developed [8]. Future studies could investigate whether such an instrument is useful in the treatment of patients with chronic somatic disorders.

Next, there is a substantial need for studies that compare blended interventions with face-to-face interventions. Only 5 studies compared a blended intervention with face-to-face care [20,24,31,33,34], which hampered drawing conclusions for this comparison. For future trials, we recommend to compare blended behavior change interventions with a control group that receives face-to-face treatment and also to include cost-effectiveness outcomes, patient satisfaction, self-management skills, attrition, or reach of the intervention. This will provide more clinically relevant information about the additional value of integrating therapeutic guidance and online care.

### Conclusions

To our knowledge, this is the first comprehensive overview of characteristics of blended behavior change interventions in patients with chronic somatic disorders. The wide variety of intervention characteristics, in terms of type and dose of therapeutic guidance, the type and dose of online care, and how these two delivery modes are integrated, hampered the investigation of intervention subtypes within the entire spectrum of blended behavior change interventions. Overall, within this heterogenic sample of studies, we found no evidence for the effectiveness of blended behavior change interventions in patients with chronic somatic disorders compared with no intervention, face-to-face behavior change interventions, or with online interventions without face-to-face support. With respect to the potential of blended behavior change interventions, we suggest investigating which type of blended intervention works for whom to come to personalized blended care for patients with chronic somatic disorders.

## Appendix

Multimedia Appendix 1 Keywords per construct (PubMed version).

Multimedia Appendix 2 Characteristics of studies, participants, and interventions.

Multimedia Appendix 3 Risk of bias assessment.

Multimedia Appendix 4 Outcome measures of studies with no intervention as control condition.

Multimedia Appendix 5 Outcome measures of studies with control conditions online behavior change intervention.

Multimedia Appendix 6 Outcome measures of studies with control conditions face-to-face behavior change intervention.

## Abbreviations

COPD: chronic obstructive pulmonary disease
RCT: randomized controlled trial
