# Supplementary material for: Blended Interventions to Change Behavior in Patients With Chronic Somatic Disorders: Systematic Review
Source: J Med Internet Res. 2017 Dec 21;19(12):e418. doi: 10.2196/jmir.8108 (PMC5754569; doi:10.2196/jmir.8108)
Supplement: Multimedia Appendix 1 [file jmir_v19i12e418_app1.pdf]

	Aspect Chronic disorder
1	“chronic disease”[MeSH Terms] OR “chronic disease”[tiab] OR “chronic diseases”[tiab] OR “chronic illness”[tiab] OR “chronic illnesses”[tiab] OR “chronic condition”[tiab] OR “chronic conditions”[tiab] OR “chronically ill”[tiab] OR disability[tiab] OR “pulmonary disease, chronic obstructive”[MeSh Terms] OR asthma[tiab] OR copd[tiab] OR “chronic obstructive pulmonary disease”[tiab] OR neoplasms[MeSh Terms] OR leukemia[MeSh Terms] OR neoplasms[tiab] OR neoplasm[tiab] OR cancer [tiab] OR leukemia[tiab] OR “coronary artery disease”[MeSh Terms] OR “coronary artery disease”[tiab] OR stroke[MeSH Terms] OR stroke[tiab] OR “heart failure”[MeSh Terms] OR “heart failure”[tiab] OR “cardiovascular disease”[tiab] OR hypertension[MeSh Terms] OR hypertension[tiab] OR “blood pressure”[MeSH Terms] OR “blood pressure”[tiab] OR “cardiovascular diseases”[MeSH Terms] OR “cardiovascular diseases” [tiab] OR “cardiovascular disease”[tiab] OR “cardiovascular risk”[tiab] OR “cardiovascular risks”[tiab] OR “peripheral vascular diseases”[MeSh Terms] OR “vascular disease”[tiab] OR “vascular diseases”[tiab] OR “liver diseases”[MeSh Terms] OR “liver diseases”[tiab] OR “liver disease”[tiab] OR “liver failure”[tiab] OR “liver dysfunction”[tiab] OR "diabetes mellitus" [MeSh Terms] OR "diabetes mellitus"[tiab] OR diabetes[tiab] OR obesity[MeSh Terms] OR obesity[tiab] OR “headache disorders”[MeSH Terms] OR “migraine disorders”[MeSh Terms] OR migraine [tiab] OR headache [tiab] OR “back pain”[MeSH Terms] OR “back pain”[tiab] OR backache[tiab] OR "arthritis, rheumatoid" [MeSh Terms] OR “musculoskeletal diseases”[MeSH Terms] OR musculoskeletal [tiab] OR arthritis [tiab] OR osteoarthritis [tiab] OR fibromyalgia [tiab] OR osteoporosis [tiab] OR rheumatism [tiab] OR arthrosis [tiab] OR “multiple sclerosis”[MeSh Terms] OR “multiple sclerosis” [tiab] OR epilepsy[MeSH Terms] OR epilepsy [tiab] OR “HIV infections” [MeSH Terms] OR aids [tiab] OR “HIV infections” [tiab] OR “immunodeficiency syndrome”[tiab] OR hiv[tiab] OR “vision disorders”[MeSH Terms] OR “vision disorders”[tiab] OR “vision disorder”[tiab] OR “hearing disorders”[MeSH Terms] OR “hearing disorders”[tiab] OR “hearing disorder”[tiab] OR “neck pain”[MeSh Terms] OR “neck pain”[tiab] OR neckache[tiab] OR “parkinson disease”[MeSH Terms] OR parkinson[tiab] OR “chronic pain”[MeSh Terms] OR somatic[tiab] OR “chronic pain”[tiab] OR “Fatigue syndrome, chronic”[MeSh Terms] OR “chronic fatigue” [tiab]
	Aspect e-Health
2	telemedicine [MeSH Terms] OR telemedicine [tiab] OR internet [MeSH Terms] OR internet [tiab] OR website [tiab] OR “world wide web”[tiab] OR web-based [tiab] OR internet-based [tiab] OR e-health [tiab] OR ehealth[tiab] OR blended [tiab] OR “smart phone” [tiab] OR “mobile health” [tiab] OR Mhealth [tiab] OR M-health [tiab] OR tele-health [tiab] OR telehealth[tiab] OR technology[tiab]
	Aspect behavior change intervention
3	Behavior[MeSH Terms] OR behavior[tiab] OR behaviour[tiab] OR behavioral[tiab] OR self-management[tiab] OR “selfmanagement”[tiab] OR lifestyle[tiab] OR behavioural[tiab] OR “behavior change”[tiab] OR “behaviour change”[tiab] OR “motor activity”[MeSH Terms] OR “physical activity”[tiab] OR “Nutrition therapy”[MeSH Terms] OR nutrition [tiab] OR dietary[tiab]
	Aspect intervention study
4	"intervention studies"[MeSH Terms] OR "intervention studies"[tiab] OR "intervention study"[tiab] OR intervention[tiab] OR experimental[tiab] OR effect[tiab] OR effectiveness[tiab] OR evaluation[tiab] OR RCT[tiab] OR CCT[tiab] OR trial[tiab] OR random*[tiab]
	Combined aspects
	#1 AND #2 AND #3 AND #4 NOT child*[tiab]
