# Supplementary material for: Blended Interventions to Change Behavior in Patients With Chronic Somatic Disorders: Systematic Review
Source: J Med Internet Res. 2017 Dec 21;19(12):e418. doi: 10.2196/jmir.8108 (PMC5754569; doi:10.2196/jmir.8108)
Supplement: Multimedia Appendix 2 [file jmir_v19i12e418_app2.pdf]

Author, year of publication	Study population (n, mean age in years, F: females [%])	Chronic disorder	Target behavior, duration of intervention	Theoretical basis	Description of blended intervention (therapeutic delivery mode and frequency; Internet-based delivery mode and frequency)	Therapeutic guidance (T[a]), online care (O[b]), and blended integration (B[c])	Type of control intervention (C)[d]
Allen et al, 2013	n=68, mean age=45, F=78%	Obesity	Weight loss (nutrition and physical activity), 6 months	Social cognitive theory	T1: 9 face-to-face sessions with counselor; T2: 7 face-to-face sessions with counselor; O: app with weekly assignments and (feedback) messages	T: b<br>O: a, d, e<br>B: b	C1: c<br>C2: b
Bennett et al, 2010	n=101, mean age=54, F=48%	Obesity and hypertension	Obesogenic behavior (individual selected), 3 months	No	T: 2 face-to-face sessions and 2 phone calls with dietitian, ask-the-expert option, and forum with peers; O: website with weekly assignments and information	T: a, b, d, g<br>O: a, b<br>B: a	C: a
De Boer et al, 2014	n=72, mean age=52, F=64%	Chronic pain	Pain thinking, feeling, and behaving; 4 months	Cognitive behavioral therapy	T: weekly email contact with a cognitive behavior therapist; O: website with weekly assignments, enriched information, and messages	T: c<br>O: a, b, c, e<br>B: a	C: b
Buhrman et al, 2004	n=56, mean age=45, F=63%	Chronic back pain	Pain thinking, feeling, and behaving, 6 weeks	Fear avoidance model	T: weekly phone calls with a therapist; O: website with weekly assignments and	T: d<br>O: a, b<br>B: a	C: a

					information		
Buhrman, 2011	n=54, mean age=43, F=69%	Chronic back pain	Pain thinking, feeling, and behaving, 11 weeks	Fear avoidance model	T: weekly email contact with psychologist; O: website with weekly assignments and messages	T: c<br>O: a, e<br>B: a	C: a
Buhrman et al, 2013	n=76, mean age=49, F=59%	Chronic pain	Pain thinking, feeling, and behaving, 7 weeks	Acceptance and commitment therapy	T: weekly email contact and 2 phone calls with clinical psychology graduate student; O: website with weekly assignments, enriched information, and messages	T: c, d<br>O: a, b, c, e<br>B: a	C: a
Buhrman et al, 2015	n=52, mean age=51, F=85%	Chronic pain	Pain thinking, feeling, and behaving, 8 weeks	Cognitive behavioral therapy	T: weekly email contact and 2 phone calls with clinical psychology graduate student; O: website with weekly assignments, information, and messages	T: c, d<br>O: a, b, e<br>B: a	C: a
Dear et al, 2015	n=490, mean age=50, F=80%	Chronic pain	Pain thinking, feeling, and behaving, 8 weeks	Cognitive behavioral therapy	T1: weekly telephone or email contact with psychologist; T2: optional telephone and email contact with psychologist; O: website with 7 assignments, information, and automatic (feedback) messages	T: c, d<br>O: a, b, d, e<br>B: a	C1: a<br>C2: c

Dlugonski, 2012	n=45, mean age=47, F=87%	Multiple sclerosis	Physical activity, 12 weeks	No	T: 7 videoconferencing sessions with health behavior coach; O: website with weekly assignments, information, and messages	T: f<br>O: a, b, e<br>B: a	C: a
Ferwerda et al, 2017	n=133, mean age=56, F=64%	Rheumatoid arthritis with heightened distress	Coping with symptoms, 9-65 weeks	Cognitive behavioral therapy	T: 1 face-to-face session with therapist, followed by weekly or biweekly email contact; O: website with assignments and psychoeducational texts	T: a, b, c<br>O: a, b, c<br>B: a	C: a
Friessen et al, 2017	n=60, mean age=48, F=95%	Fibromyalgia	Coping with pain, 8 weeks	Cognitive behavioral therapy	T: weekly telephone contact with trained student and email contact; O: website with weekly assignments, enriched information, and automated messages	T: c, d<br>O: a, b, c, e<br>B: a	C: a
Glasgow et al, 2010	n=463, mean age=58, F=50%	Diabetes Mellitus type II	Nutrition, physical activity, and medication taking, 4 months	Social ecological theory, 5 A's self-management model	T: 2 phone calls with therapist, 3 group sessions, and forum with peers; O: website with weekly assignments, enriched information, and automatic telephonic (feedback) messages	T: b, d, g<br>O: a, b, c, e<br>B: a	C: c
Hunt et al, 2009	n=54, mean age=39, F=80%	Irritable bowel syndrome	Stress management and catastrophic thinking, 5 weeks	Cognitive behavioral therapy	T: weekly email contact with therapist; O: website with	T: c<br>O: a, b<br>B: a	C: a

					weekly assignments and information		
Jasper et al, 2014	n=128, mean age=51, F=40%	Chronic tinnitus	Relaxation, positive thinking, and cognitive restructuring, 10 weeks	Cognitive behavioral therapy	T: weekly chat session and email contact with cognitive behavior therapist; O: website with weekly assignments and information	T: a, c<br>O: a, b<br>B: b	C1: b<br>C2: a
Klaren et al, 2014	n=70, mean age=50, F=78%	Multiple sclerosis	Sedentary behavior, 6 months	Social cognitive theory	T: 13 videoconferences with coach; O: website with 13 assignments and enriched information	T: f<br>O: a, b, c<br>B: a	C: a
Liebreich, Plotnikoff et al, 2009	n=49, mean age=54, F=59%	Diabetes Mellitus type II	Physical activity, 12 weeks	Social cognitive theory	T: weekly email contact with counselor and ask-the-expert option, and forum with peers; O: website with weekly assignments and information	T: a, c, g<br>O: a, b<br>B: a	C: c
Ljotsson, Hedman et al, 2011	n=61, mean age=35, F=74%	Irritable bowel syndrome	Avoidance behavior, 10 weeks	Acceptance and commitment therapy	Cognitive behavioral intervention; T: weekly email contact with psychologist, ask-the-expert option, and forum with peers; O: website with weekly assignments and information	T: a, c, g<br>O: a, b<br>B: a	C: a
McKay et al, 2001	n=78, mean age=52, F=53%	Diabetes Mellitus type II	Physical activity, 8 weeks	Multilevel social ecological model of diabetes self-management	Behavioral intervention to increase physical activity; T: weekly email contact with occupational therapist,	T: a, c, g<br>O: a, b, c, d<br>B: a	C: c

					ask-the-expert option, and forum with peers; O: website with weekly assignments		
Moss Morris et al, 2012	n=40, mean age=41, F=80%	Multiple sclerosis	Fatigue behavior, 10 weeks	Cognitive behavioral therapy	T: 3 phone sessions of 30-60 min with assistant psychologist; O: website with weekly assignments and information	T: d<br>O: a, b, c, e<br>B: a	C: a
Nobis et al, 2015	n=256, mean age=51, F=63%	Diabetes Mellitus type I and II	Depressive symptoms, 8 weeks	Systematic behavioral activation and problem solving	T: weekly email or phone contact with psychologist or graduate student; O: website with weekly assignments, information, and automatic text messages	T: a, c, d, e<br>O: a, b, e<br>B: a	C: c
Nordin, et al, 2016	n=99, mean age=43, F=85%	Persistent musculoskeletal pain	Pain behavior, 4 months	Cognitive behavioral therapy	T: treatments from at least three different occupations according to an individualized treatment plan; O: website with assignments, enriched information, and automated messages	T: b<br>O: a, b, c, e<br>B: b	C: b
Steel et al, 2016	n=261, mean age=61, F=27%	Advanced cancer	Pain, fatigue, and depression, 6 months	Cognitive behavioral therapy	T: email contact every 2 weeks, face-to-face contact with care coordinator every 2 months, and forum with peers; O: website with assignments and enriched information	T: a, b, c, g<br>O: a, b, c<br>B: b	C: a

Torbjørnsen et al, 2014	n=151, mean age=57, F=41%	Diabetes Mellitus type II	Nutrition and physical activity, 4 months	No	T: 5 phone sessions with a diabetes specialist nurse and ask-the-expert option; O: app with daily assignment, enriched information, and (feedback) messages	T: a, d<br>O: a, b, c, d, e<br>B: b	C1: c<br>C2: a
Trompetter et al, 2015	n=238, mean age=53, F=76%	Chronic pain	Pain behavior, 3 months	Acceptance and commitment therapy	T: weekly email contact with counselor; O: website with weekly assignments and enriched information	T: c<br>O: a, b, c<br>B: a	C1: c<br>C2: a
van Beugen et al, 2016	n=131, mean age=53, F=49%	Psoriasis	Pain, fatigue, and negative mood, 25 weeks	Cognitive behavioral therapy	T: 2 face-to-face sessions with therapist, telephonic instruction, and weekly feedback emails; O: website with information and assignments	T: b, c, d<br>O: a, b<br>B: b	C: a
van der Berg et al, 2006	n=160, mean age=50, F=76%	Rheumatoid arthritis	Physical activity, 12 months	No	T: weekly email contact with physical therapist, 4 group meetings, and forum with peers; O: website with weekly assignments	T: b, c, g<br>O: a<br>B: b	C: c
Van der Meer et al, 2009	n=200, mean age=31, F=69%	Asthma	Asthma control: measuring FEV1 and inhaler technique, 12 months	No	T: 1 group session and optional phone calls and email contact with a respiratory nurse; O: website with weekly assignments, enriched information, and automatic (feedback)	T: a, b, c, d, e<br>O: a, b, d, e<br>B: a	C: a

					messages		
Van der Weegen et al, 2015	n=199, mean age=58, F=51%	COPD or Diabetes Mellitus type II	Physical activity, 4-6 months	No	T: 4 face-to-face sessions with nurse and mail contact, and option to request for advice; O: activity tracker, mobile app and website with assignments, and automated tailored feedback messages	T: a, b, c<br>O: a, d, e<br>B: a	C1: a<br>C2: b
Yardley et al, 2014	n=179, mean age=51, F=66%	Obesity	Weight management and nutrition, 6 months	Cognitive behavioral therapy	T1: 3 face-to-face, phone, or email contacts with a nurse and ask-the-expert option; T2: 7 face-to-face, phone, or email contacts with a nurse and ask-the-expert option; O: website with weekly assignments, enriched information, and (feedback) messages	T: a, b, c, d<br>O: a, b, c, d, e<br>B: a	C1: a<br>C2: c

[a]T: therapeutic guidance: (1) option to request for advice, (2) face-to-face contact, (3) email, (4) phone calls, (5) short messaging service, (6) videoconferencing, and (7) discussion forum with peers.

[b]O: online care: (1) assignments, (2) information, (3) enriched information environment, (4) automated tailored feedback, and (5) automated messages.

[c]B: blended integration: (1) online care and therapeutic guidance are integrated and (2) online care and professionals are two separate elements.

[d]C: control conditions: (1) no intervention, (2) face-to-face behavior change interventions, and (3) online behavior change intervention.
