# Supplementary material for: Blended Interventions to Change Behavior in Patients With Chronic Somatic Disorders: Systematic Review
Source: J Med Internet Res. 2017 Dec 21;19(12):e418. doi: 10.2196/jmir.8108 (PMC5754569; doi:10.2196/jmir.8108)
Supplement: Multimedia Appendix 3 [file jmir_v19i12e418_app3.pdf]

Author, year of publication	Random sequence generation	Allocation concealment	Blinding of outcome assessor	Incomplete outcome data	Selective reporting	Group similarity at baseline	Cointer-vention	Comp-liance	Intention-to-treat analysis	Timing outcome assess-ments	Score (0-10)[a]	Total: low or high quality
Allen et al, 2013 [37]	L[b]	U[c]	U	L	L	L	U	L	H[d]	L	6	High
Bennett et al, 2010 [38]	L	U	U	L	L	L	U	L	L	L	7	High
De Boer et al, 2014 [20]	L	L	U	L	L	U	U	L	L	L	7	High
Buhrman et al, 2004 [21]	L	U	U	L	L	L	U	U	H	L	5	Low
Buhrman, 2011 [23]	L	U	U	L	L	L	U	U	L	L	6	High
Buhrman et al, 2013 [22]	L	L	U	L	L	L	U	U	L	L	7	High
Buhrman et al, 2015 [24]	L	L	U	L	L	L	U	U	L	L	7	High
Dear et al, 2015 [25]	L	L	U	L	L	L	U	U	U	L	6	High
Dlugonski et al, 2012 [45]	L	H	U	L	L	L	U	L	L	L	7	High

Ferwerda et al, 2017 [33]	L	L	U	H	L	L	U	H	L	L	6	High
Friessen et al, 2017 [34]	L	L	H	L	L	L	U	L	L	L	8	High
Glasgow et al, 2010 [40]	L	U	U	L	L	L	U	L	L	L	7	High
Hunt et al, 2009 [28]	L	H	U	H	L	L	U	U	H	L	4	Low
Jasper et al, 2014 [30]	L	L	U	L	L	L	U	L	L	L	8	High
Klaren et al, 2014 [46]	L	L	U	U	H	H	U	U	L	L	4	Low
Liebreich et al, 2009 [41]	L	U	U	L	L	H	U	L	L	L	6	High
Ljotsson et al, 2011 [29]	L	L	U	L	L	L	U	L	L	L	8	High
McKay et al, 2001 [42]	L	L	U	L	L	L	U	L	L	L	8	High
Moss Morris et al, 2012 [32]	L	U	U	L	L	H	L	L	L	L	7	High
Nobis et al, 2015 [31]	L	U	U	L	L	L	U	L	L	L	7	High
Nordin et al, 2016 [27]	L	L	U	L	H	L	U	L	L	L	7	High

Steel et al, 2016 [36]	L	L	L	L	L	L	U	L	L	L	9	High
Torbjørnsen et al, 2014 [43]	L	L	U	L	H	L	U	U	L	L	6	High
Trompetter et al, 2015 [26]	L	U	U	L	L	L	U	L	L	L	7	High
van Beugen et al, 2016 [35]	L	L	U	H	L	L	U	L	L	L	7	High
van der Berg et al, 2006[47]	L	L	L	L	L	L	L	L	L	L	10	High
Van der Meer et al, 2009 [48]	L	H	U	H	L	L	U	U	H	L	4	Low
Van der Weegen et al, 2015 [44]	L	L	L	L	L	H	U	U	L	L	7	High
Yardley et al, 2014 [39]	L	H	H	L	H	L	H	L	L	L	6	High

[a]Total score: low risk=1 point; unclear or high risk=0 point; low quality=0-5 points, high quality=6 points or more.

[b]L: low risk.

[c]U: unclear.

[d]H: high risk.
