# Supplementary material for: Blended Interventions to Change Behavior in Patients With Chronic Somatic Disorders: Systematic Review
Source: J Med Internet Res. 2017 Dec 21;19(12):e418. doi: 10.2196/jmir.8108 (PMC5754569; doi:10.2196/jmir.8108)
Supplement: Multimedia Appendix 4 [file jmir_v19i12e418_app4.pdf]

Author, year of publication	Outcome measure	Instrument	Time of measurement (pre- and post-treatment)	Pre-treatment mean (SD)	Post-treatment mean (SD)	Significant effect
**Symptoms and Signs**						
Bennett, 2010	Body weight	Weighing scale	0, 12 weeks	I:101.0 (15.4)<br>C: 97.3 (10.9)	I: 98.7 (3.2)<br>C: 97.6 (0.6)	No
	BMI	Scale and meter	0, 12 weeks	I: 35.0 (3.5)<br>C: 34.6 (3.2)	I: 34.1 (1.2)<br>C: 34.5 (0.8)	No
Buhrman, 2004	Pain	Scale 0-100, average score of 3 daily assessments	0, 8 weeks	I: 37.4 (18.2)<br>C: 44.4 (14.2)	I: 34.3 (16.8)<br>C: 39.6 (16.3)	No
	Pain	Multidimensional Pain Inventory: subscale pain severity	0, 8 weeks	I: 3.8 (1.9)<br>C: 5.0 (1.7)	I: 2.4 (1.1)<br>C: 3.2 (0.8)	No
Buhrman, 2011	Pain	Multidimensional Pain Inventory: subscale pain severity	0, 12 weeks	I: 3.5 (2.5)<br>C: 3.2 (2.2)	I: 3.2 (2.2)<br>C: 3.4 (2.6)	No
Buhrman, 2013	Pain	Multidimensional Pain Inventory: subscale pain severity	0, 8 weeks	I: 4.51 (0.80)<br>C: 4.35 (0.88)	I: 4.30 (1.04)<br>C: 4.29 (1.00)	No
Buhrman, 2015	Pain	Multidimensional Pain Inventory: subscale pain severity	0, 8 weeks	I: 3.81 (1.14)<br>C: 3.90 (0.89)	I: 3.75 (1.05)<br>C: 3.95 (0.93)	No
Dear, 2015 *regular contact group	Pain	Wilcinson Brief Pain Questionnaire	0, 8 weeks	I: 5.74 (1.72)<br>C: 5.98 (1.53)	I: 4.86 (1.79)<br>C: 5.71 (1.50)	Yes
Dear, 2015 *optional contact group	Pain	Wilcinson Brief Pain Questionnaire	0, 8 weeks	I: 5.54 (1.74)<br>C: 5.98 (1.53)	I: 4.85 (1.73)<br>C: 5.71 (1.50)	Yes
Ferwerda, 2017	Pain	Impact of Rheumatic Diseases on General Health and Lifestyle	0, post-intervention	I: 14.78 (4.76)<br>C: 15.77 (3.88)	I: 14.60 (4.50)<br>C: 15.68 (3.73)	No
	Fatigue	Checklist Individual Strength	0, post-intervention	I: 35.98 (11.49)<br>C: 38.24 (10.06)	I: 32.13 (11.46)<br>C: 35.88 (10.71)	No

	Disease activity	Rheumatoid Arthritis Disease Activity Index	0, post-intervention	I: 3.31 (1.99)<br>C: 3.84 (1.75)	I: 3.19 (2.10)<br>C: 3.62 (1.68)	No
Friessen, 2017	Severity and symptoms of fibromyalgia	Revised Fibromyalgia Impact Questionnaire	0, 8 weeks	I: 65.19 (13.07)<br>C: 67.75 (13.51)	I: 53.39 (18.94)<br>C: 64.59 (12.75)	Yes
	Pain	Brief Pain Inventory – subscale pain severity	0, 8 weeks	I: 5.45 (1.10)<br>C: 6.02 (1.39)	I: 4.99 (1.66)<br>C: 6.28 (1.28)	Yes
	Fatigue	Fatigue Symptom Inventory	0, 8 weeks	I: 44.30 (12.83)<br>C: 49.20 (14.29)	I: 39.69 (15.10)<br>C:47.87 (12.75)	No
Hunt, 2009	Abdominal symptoms: pain, bloating, constipation, diarrhea and satiety	Gastrointestinal Symptom Rating Scale	0, 5 weeks	I: 57 (13)<br>C: 61 (14)	I: 35 (12)<br>C: 52 (14)	Yes
Ljotsson, 2011	Abdominal symptoms: pain, bloating, constipation, diarrhea and satiety	Gastrointestinal Symptom Rating Scale	0, 10 weeks	I: 44.6 (11.1)<br>C: 39.8 (12.0)	I: 31.0 (10.2)<br>C: 40.9 (14.5)	Yes
Van der Meer, 2009	Forced expiratory volume in 1 second (FEV1)	Handheld electronic spirometer	0, 12 months	I: 3.08 (NM)<br>C: 3.13 (NM)	I: 3.32 (NM)<br>C: 3.12 (NM)	Yes
Steel, 2016	Fatigue	Functional Assessment of Cancer Therapy-Fatigue	0, 6 months	I: 36.8 (7.9)<br>C: 35.4 (7.1)	I: 28.3 (9.4)<br>C: 31.1 (11.4)	No
	Pain and impact on functioning	Brief Pain Inventory	0, 6 months	I: 5.8 (1.2)<br>C: 6.1 (1.6)	I: 4.7 (1.5)<br>C: 6.1 (2.6)	No
Torbjørnsen, 2014	Hemoglobin	DCA Vantage Analyzer	0, 4 months	I: 8.2 (1.08)<br>C: 8.3 (1.08)	I: 7.8 (1.44)<br>C: 8.0 (1.44)	No
Trompetter, 2015	Pain	Pain NRS	0, 3 months	I: 6.3 (1.6)<br>C: 6.2 (1.6)	I: 5.4 (2.2)<br>C: 5.6 (2.1)	No
Van Beugen, 2016	Fatigue	Checklist Individual Strength	0, post-treatment	I: 37.70 (10.68)<br>C: 34.31 (10.30)	I: 30.77 (12.46)<br>C: 33.8 (9.19)	Yes
	Itch	Impact of chronic Skin Disease on	0, post-treatment	I: 9.44 (3.50)<br>C: 9.06 (3.75)	I: 7.09 (3.51)<br>C: 7.44 (3.67)	No

		daily Life – itch subscale				
	Psoriasis severity – clinician rated	Psoriasis Area and Severity Index	0, post-treatment	I: 5.99 (5.61)<br>C: 4.20 (2.87)	I: 5.04 (4.59)<br>C: 3.79 (2.94)	No
	Psoriasis severity – self-reported	Psoriasis Area and Severity Index	0, post-treatment	I: 5.27 (3.29)<br>C: 4.48 (2.41)	I: 4.61 (5.39)<br>C: 3.95 (2.26)	No
Yardley, 2014 *basic nurse support	Body weight	Weighing scale	0, 6 months	I: 103.43 (25.23)<br>C: 103.27 (20.09)	I: 97.92 (5.80)<br>C: 99.38 (5.75)	No
Yardley, 2014 *regular nurse support	Body weight	Weighing scale	0, 6 months	I: 100.36 (19.38)<br>C: 103.27 (20.09)	I: 97.14 (5.74)<br>C: 99.38 (5.75)	No
**Limitations in daily activities**						
Buhrman, 2015	Disruption of daily activities by chronic pain	Pain Disability Index	0, 8 weeks	I: 36.71 (9.96)<br>C: 36.96 (10.25)	I: 32.13 (9.64)<br>C: 36.65 (9.91)	Yes
Dear, 2015 *regular contact group	Backpain associated disability in daily activities	Roland-Morris Disability Questionnaire	0, 8 weeks	I: 13.47 (5.23)<br>C: 13.93 (5.22)	I: 11.05 (5.63)<br>C: 13.97 (5.17)	Yes
Dear, 2015 *optional contact group	Backpain associated disability in daily activities	Roland-Morris Disability Questionnaire	0, 8 weeks	I: 13.24 (5.60)<br>C: 13.93 (5.22)	I: 10.95 (5.84)<br>C: 13.97 (5.17)	Yes
Dlugonski, 2012	Walking mobility	Multiple Sclerosis Walking Scale-12	0, 3 months	I: 27.4 (22.0)<br>C: 24.9 (25.0)	I: 30.9 (22.1)<br>C: 27.0 (25.6)	No
	Physical activity	Godin Leisure-Time Exercise Questionnaire	0, 3 months	I: 13.6 (11.6)<br>C: 16.1 (14.2)	I: 28.2 (15.6)<br>C: 15.4 (13.9)	Yes
Ferwerda, 2017	Self-care	Impact of Rheumatic Diseases on General Health and Lifestyle	0, post-intervention	I: 27.39 (5.72)<br>C: 25.04 (6.52)	I: 27.35 (5.94)<br>C: 24.41 (6.92)	No
	Mobility	Impact of Rheumatic Diseases on General Health and Lifestyle	0, post-intervention	I: 22.00 (5.78)<br>C: 19.82 (6.40)	I: 22.30 (5.55)<br>C: 19.37 (6.55)	No
Jasper, 2014	Insomnia	Insomnia Severity index	0, 10 weeks	I: 12.68 (5.91)<br>C: 11.25 (6.51)	I: 8.70 (5.80)<br>C: 10.91 (7.21)	Yes
Klaren, 2014	Sedentary behavior	International Physical Activity Questionnaire	0, 6 months	I: 550 (233)<br>C: 412 (193)	I: 429.2 (201.2)<br>C: 528.2 (200.7)	Yes
Ljotsson, 2011	Functional	Sheehan	0, 10 weeks	I: 11.9 (8.1)	I: 8.7 (6.3)	Yes

	impairment in work/school, social life and family	Disability Scales		C: 6.4 (6.7)	C: 7.8 (7.6)	
Trompetter, 2015	Disruption of daily activities by chronic pain	Pain Disability Index	0, 3 months	I: 36.0 (12.7)<br>C: 36.1 (12.7)	I: 30.6 (14.5)<br>C: 33.0 (14.0)	No
Van der Weegen, 2015	Physical activity	Pam accelerometer	0, post-intervention	I: 39.29 (18.1)<br>C: 44.13 (20.3)	I: 48.16 (23.8)<br>C: 39.16 (19.5	Yes
**Dealing with the chronic condition: cognitive measures**						
Buhrman, 2004	Coping strategy: diverting attention	Coping Strategy Questionnaire: subscale diverting attention	0, 8 weeks	I: 11.6 (5.7)<br>C: 12.3 (7.4)	I: 12.3 (5.2)<br>C: 11.9 (6.9)	No
	Coping strategy: reinterpret pain sensation	Coping Strategy Questionnaire: subscale reinterpret pain sensation	0, 8 weeks	I: 3.6 (3.5)<br>C: 5.4 (6.5)	I: 4.4 (3.6)<br>C: 4.6 (5.9	No
	Coping strategy: coping self-statements	Coping Strategy Questionnaire: subscale coping self-statements	0, 8 weeks	I: 18.4 (6.5)<br>C: 18.3 (6.6)	I: 19.1 (5.8)<br>C: 17.3 (6.7)	No
	Coping strategy: ignore pain sensations	Coping Strategy Questionnaire: subscale ignore pain sensations	0, 8 weeks	I: 13.1 (13.7)<br>C: 13.5 (6.6)	I: 13.7 (7.0)<br>C: 12.9 (6.5)	No
	Coping strategy: praying or hoping	Coping Strategy Questionnaire: subscale praying or hoping	0, 8 weeks	I: 12.0 (6.9)<br>C: 10.4 (6.7)	I: 9.8 (5.1)<br>C: 8.5 (6.0)	No
	Coping strategy: catastrophizin g	Coping Strategy Questionnaire: subscale catastrophizing	0, 8 weeks	I: 13.6 (7.7)<br>C: 13.7 (6.9)	I: 8.6 (5.2)<br>C: 12.3 (7.2)	Yes
	Coping strategy: control over pain	Coping Strategy Questionnaire: subscale control over pain	0, 8 weeks	I: 2.8 (1.0)<br>C: 2.9 (1.1)	I: 3.9 (0.7)<br>C: 2.9 (1.0)	Yes
	Coping strategy: ability to decrease pain	Coping Strategy Questionnaire: subscale ability to decrease pain	0, 8 weeks	I: 3.0 (0.8)<br>C: 2.6 (1.0)	I: 3.9 (0.9)<br>C: 2.9 (1.0)	Yes
	Perceived life control	Multidimensional Pain Inventory: subscale life control	0, 8 weeks	I: 3.1 (1.1)<br>C:2.7 (0.9)	I: 3.9 (1.0)<br>C: 3.1 (0.9)	No
	Perception of support received from	Multidimensional Pain Inventory: subscale	0, 8 weeks	I: 4.0 (1.6)<br>C: 3.9 (1.5)	I: 4.2 (1.3)<br>C: 3.8 (1.6)	No

	others					
	Perception of received punishing responses	Multidimensional Pain Inventory: subscale punishing responses	0, 8 weeks	I: 1.0 (1.4)<br>C:1.5 (1.4)	I: 0.7 (1.1)<br>C: 1.2 (1.3)	No
	Perception of received solicitous responses	Multidimensional Pain Inventory: subscale solicitous responses	0, 8 weeks	I: 2.3 (1.4)<br>C: 2.1 (1.4)	I: 2.3 (1.2)<br>C: 1.9 (1.5)	No
	Perception of received distracting responses	Multidimensional Pain Inventory: subscale distracting responses	0, 8 weeks	I: 2.5 (1.7)<br>C: 2.7 (1.7)	I: 2.5 (1.6)<br>C: 2.5 (1.7)	No
Buhrman, 2011	Beliefs and attitudes associated with chronic pain	Pain and Impairment Relationship Scale	0, 12 weeks	I: 53.3 (10.4)<br>C: 48.3 (13.7)	I: 49.1 (11.0)<br>C: 46.1 (18.7)	No
	Coping strategy: diverting attention	Coping Strategy Questionnaire: subscale diverting attention	0, 12 weeks	I: 11.2 (5.9)<br>C: 11.4 (5.7)	I: 11.5 (6.5)<br>C: 10.8 (5.5)	No
	Coping strategy: reinterpret pain sensation	Coping Strategy Questionnaire: subscale reinterpret pain sensation	0, 12 weeks	I: 5.3 (5.2)<br>C: 5.4 (3.9)	I: 6.2 (4.5)<br>C: 6.1 (5.1)	No
	Coping strategy: coping self-statements	Coping Strategy Questionnaire: subscale coping self-statements	0, 12 weeks	I: 21.0 (5.9)<br>C: 18.3 (6.7)	I: 19.1 (7.6)<br>C: 19.4 (7.5)	No
	Coping strategy: ignore pain sensations	Coping Strategy Questionnaire: subscale ignore pain sensations	0, 12 weeks	I: 15.4 (6.0)<br>C: 15.3 (7.0)	I: 17.6 (7.7)<br>C: 14.7 (7.4)	No
	Coping strategy: praying or hoping	Coping Strategy Questionnaire: subscale praying or hoping	0, 12 weeks	I: 11.0 (7.4)<br>C: 10.8 (5.9)	I: 10.8 (7.0)<br>C: 9.2 (5.9)	No
	Coping strategy: catastrophizing	Coping Strategy Questionnaire: subscale catastrophizing	0, 12 weeks	I: 14.3 (6.1)<br>C: 12.0 (8.2)	I: 9.5 (5.5)<br>C: 11.6 (8.2)	Yes
	Coping strategy: control over pain	Coping Strategy Questionnaire: subscale control over pain	0, 12 weeks	I: 3.3 (1.3)<br>C: 2.9 (1.4)	I: 3.0 (1.1)<br>C:2.8 (1.5)	No
	Coping	Coping Strategy	0, 12 weeks	I: 3.1 (0.9)	I: 3.3 (0.8)	No

	strategy: ability to decrease pain	Questionnaire: subscale ability to decrease pain		C: 3.0 (1.0)	C: 3.0 (1.2)	
	Perceived life control	Multidimensional Pain Inventory: subscale life control	0, 12 weeks	I: 3.1 (1.1)<br>C: 2.7 (0.9)	I: 3.9 (1.0)<br>C: 3.1 (0.9)	No
	Perception of support received from others	Multidimensional Pain Inventory: subscale	0, 12 weeks	I: 4.0 (1.6)<br>C: 3.9 (1.5)	I: 4.2 (1.3)<br>C: 3.8 (1.6)	No
	Perception of received punishing responses	Multidimensional Pain Inventory: subscale punishing responses	0, 12 weeks	I: 1.0 (1.4)<br>C: 1.5 (1.4)	I: 0.7 (1.1)<br>C:1.2 (1.3)	No
	Perception of received solicitous responses	Multidimensional Pain Inventory: subscale solicitous responses	0, 12 weeks	I: 2.3 (1.4)<br>C: 2.1 (1.4)	I: 2.3 (1.2)<br>C: 1.9 (1.5)	No
	Perception of received distracting responses	Multidimensional Pain Inventory: subscale distracting responses	0, 12 weeks	I: 2.5 (1.7)<br>C: 2.7 (1.7)	I: 2.5 (1.6)<br>C: 2.5 (1.7)	No
Buhrman, 2013	Beliefs and attitudes associated with chronic pain	Pain And Impairment Relationship Scale	0, 8 weeks	I: 60.89 (10.27)<br>C: 59.79 (9.93)	I: 55.88 (12.43)<br>C: 59.24 (10.29)	No
	Coping strategy: diverting attention	Coping Strategy Questionnaire: subscale diverting attention	0, 8 weeks	I: 15.01 (6.15)<br>C: 14.87 (6.10)	I: 14.54 (6.08)<br>C: 14.76 (6.48)	No
	Coping strategy: reinterpret pain sensation	Coping Strategy Questionnaire: subscale reinterpret pain sensation	0, 8 weeks	I: 9.03 (5.65)<br>C:8.29 (5.57)	I: 9.39 (6.97)<br>C: 7.88 (5.25)	No
	Coping strategy: coping self-statements	Coping Strategy Questionnaire: subscale coping self-statements	0, 8 weeks	I: 17.79 (5.58)<br>C: 18.18 (6.43)	I: 18.66 (6.23)<br>C: 19.20 (6.24)	No
	Coping strategy: ignore pain sensations	Coping Strategy Questionnaire: subscale ignore pain sensations	0, 8 weeks	I: 13.66 (5.85)<br>C: 14.26 (5.20)	I: 14.40 (5.59)<br>C: 15.50 (5.33)	No
	Coping strategy: praying or	Coping Strategy Questionnaire: subscale praying	0, 8 weeks	I: 12.00 (7.42)<br>C: 11.34 (6.73)	I: 11.93 (7.96)<br>C: 13.96 (6.33)	Yes

	hoping	or hoping				
	Coping strategy: catastrophizing	Coping Strategy Questionnaire: subscale catastrophizing	0, 8 weeks	I: 17.82 (5.78)<br>C: 18.58 (6.42)	I: 16.08 (5.91)<br>C: 19.00 (5.56)	Yes
	Perceived life control	Multidimensional Pain Inventory: subscale life control	0, 8 weeks	I: 2.62 (0.96)<br>C: 2.41 (1.21)	I: 2.96 (1.67)<br>C: 2.54 (1.22)	No
	Perception of support received from others	Multidimensional Pain Inventory: subscale	0, 8 weeks	I: 4.06 (1.55)<br>C: 4.11 (1.38)	I: 3.69 (1.66)<br>C: 4.05 (1.33)	No
	Perception of received punishing responses	Multidimensional Pain Inventory: subscale punishing responses	0, 8 weeks	I: 2.17 (1.38)<br>C: 2.25 (1.52)	I: 1.84 (1.49)<br>C: 2.15 (1.40)	No
	Perception of received solicitous responses	Multidimensional Pain Inventory: subscale solicitous responses	0, 8 weeks	I: 2.72 (1.60)<br>C:2.73 (1.25)	I: 2.44 (1.64)<br>C: 2.72 (1.34)	No
	Perception of received distracting responses	Multidimensional Pain Inventory: subscale distracting responses	0, 8 weeks	I: 2.55 (1.36)<br>C: 2.19 (1.03)	I: 2.60 (1.43)<br>C: 2.18 (1.16)	No
Buhrman, 2015	Fear of symptoms	Anxiety Sensitivity Index	0, 8 weeks	I: 24.57 (2.61)<br>C:18.87 (8.16)	I: 18.90 (12.23)<br>C: 17.37 (7.50)	No
	Pain-related catastrophizing	Pain Catastrophizing Scale	0, 8 weeks	I: 22.71 (8.58)<br>C: 24.58 (9.20)	I: 14.49 (9.49)<br>C:22.94 (11.65)	Yes
	Coping strategy: diverting attention	Coping Strategy Questionnaire: subscale diverting attention	0, 8 weeks	I: 13.69 (5.06)<br>C: 11.87 (5.41)	I:13.07 (6.09)<br>C: 12.41 (4.84)	No
	Coping strategy: reinterpret pain sensation	Coping Strategy Questionnaire: subscale reinterpret pain sensation	0, 8 weeks	I: 6.25 (5.43)<br>C: 6.54 (6.09)	I: 7.10 (6.22)<br>C: 5.75 (5.73)	No
	Coping strategy: coping self-statements	Coping Strategy Questionnaire: subscale coping self-statements	0, 8 weeks	I: 17.25 (5.68)<br>C: 16.70 (5.00)	I: 19.04 (6.63)<br>C: 18.86 (6.08)	No
	Coping strategy: ignore pain sensations	Coping Strategy Questionnaire: subscale ignore pain sensations	0, 8 weeks	I: 13.86 (8.98)<br>C: 14.96 (6.13)	I: 15.36 (7.64)<br>C: 16.73 (5.84)	No
	Coping	Coping Strategy	0, 8 weeks	I: 9.68 (6.56)	I: 10.77 (7.49)	No

	strategy: praying or hoping	Questionnaire: subscale praying or hoping		C:10.58 (7.91)	C: 9.62 (8.00)	
	Coping strategy: catastrophizing	Coping Strategy Questionnaire: subscale catastrophizing	0, 8 weeks	I: 16.36 (7.12)<br>C: 17.83 (6.84)	I: 10.06 (6.35)<br>C: 16.36 (5.64)	Yes
	Perceived life control	Multidimensional Pain Inventory: subscale life control	0, 8 weeks	I: 2.41 (0.87)<br>C: 2.51 (0.79)	I: 3.48 (1.42)<br>C: 2.94 (0.74)	No
	Perception of support received from others	Multidimensional Pain Inventory: subscale	0, 8 weeks	I: 3.36 (1.57)<br>C:3.61 (1.36)	I: 3.26 (1.70)<br>C: 3.89 (1.31)	No
	Perception of received punishing responses	Multidimensional Pain Inventory: subscale punishing responses	0, 8 weeks	I: 2.05 (1.51)<br>C: 2.10 (1.73)	I: 2.13 (1.72)<br>C: 2.13 (1.72)	No
	Perception of received solicitous responses	Multidimensional Pain Inventory: subscale solicitous responses	0, 8 weeks	I: 2.57 (1.56)<br>C:2.41 (1.30)	I: 2.17 (1.44)<br>C: 2.50 (1.38)	No
	Perception of received distracting responses	Multidimensional Pain Inventory: subscale distracting responses	0, 8 weeks	I: 3.42 (5.35)<br>C:2.29 (1.16)	I: 2.41 (1.05)<br>C: 2.48 (1.30)	No
Dear, 2015 *regular contact group	Pain self-efficacy	Pain Self-efficacy Questionnaire	0, 8 weeks	I: 28.86 (12.93)<br>C: 28.63 (12.10)	I: 35.94 (12.98)<br>C: 29.68 (12.11)	Yes
	Fear of movement	TAMPA Scale of Kinesiophobia	0, 8 weeks	I: 38.59 (7.99)<br>C: 39.56 (8.67)	I: 34.48 (7.00)<br>C: 37.65 (8.60)	Yes
	Acceptance of pain	Chronic Pain Acceptance Questionnaire	0, 8 weeks	I: 22.58 (7.53)<br>C: 22.67 (7.52)	I: 26.79 (6.69)<br>C: 23.66 (7.66)	Yes
Dear, 2015 *optional contact group	Pain self-efficacy	Pain Self-efficacy Questionnaire	0, 8 weeks	I: 28.61 (14.08)<br>C: 28.63 (12.10)	I: 33.60 (13.83)<br>C: 29.68 (12.11)	Yes
	Fear of movement	TAMPA Scale of Kinesiophobia	0, 8 weeks	I: 37.60 (8.40)<br>C:39.56 (8.67)	I: 34.88 (7.80)<br>C: 37.65 (8.60)	Yes
	Acceptance of pain	Chronic Pain Acceptance Questionnaire	0, 8 weeks	I: 23.30 (7.72)<br>C: 22.67 (7.52)	I: 25.40 (8.01)<br>C: 23.66 (7.66)	No
Friessen, 2017	Fear of movement	TAMPA Scale of Kinesiophobia	0, 8 weeks	I: 38.35 (7.39)<br>C: 40.57 (5.55)	I: 33.87 (6.25)<br>C: 42.73 (4.64)	No
	Pain self-efficacy	Pain Self-Efficacy Questionnaire	0, 8 weeks	I: 22.93 (9.78)<br>C: 19.83	I: 29.99 (11.10)<br>C: 22.00 (10.18)	No

				(10.25)		
	Pain coping	Pain Responses Self-Statements: subscale coping	0, 8 weeks	I: 29.73 (5.94)<br>C: 23.77 (8.22)	I: 31.99 (5.72)<br>C: 23.75 (7.74)	No
	Pain catastrophizing	Pain Responses Self-Statements: subscale catastrophizing	0, 8 weeks	I: 21.52 (7.37)<br>C: 24.53 (9.77)	I: 17.23 (9.26)<br>C: 24.09 (9.51)	No
Hunt, 2009	Catastrophizing	Consequences of Physical Sensations Questionnaire	0, 5 weeks	I: 2.1 (0.51)<br>C: 2.1 (0.57)	I: 1.25 (0.39)<br>C: 2.1 (0.56)	Yes
Jasper, 2014	Acceptance	Tinitis Acceptance Questionnaire	0, 10 weeks	I: 42.07 (11.70)<br>C: 42.84 (13.48)	I: 47.91 (11.70)<br>C: 43.99 (13.51)	Yes
Ljotsson, 2011	Fear of irritable bowel syndrome symptoms	Visceral Sensitivity Index	0, 10 weeks	I: 32.5 (18.0)<br>C: 27.5 (16.3)	I: 14.1 (15.1)<br>C: 26.2 (17.9)	Yes
Moss Morris, 2012	The impact of fatigue on patient's daily life	Modified fatigue impact scale	0, 10 weeks	I: 13.17 (3.81)<br>C: 12.69 (3.89)	I: 9.00 (3.75)<br>C: 12.88 (3.89)	Yes
Trompetter, 2015	Mindfulness skills	Five Facet Mindfulness Questionnaire-Short Form	0, 3 months	I: 81.4 (10.7)<br>C: 80.4 (10.7)	I: 86.7 (12.2)<br>C: 83.3 (11.7)	No
	Avoidance of pain and cognitive fusion with pain	Psychological Inflexibility in Pain Scale	0, 3 months	I: 55.0 (11.6)<br>C: 54.5 (11.6)	I: 40.7 (13.8)<br>C: 48.8 (13.1)	Yes
	Pain catastrophizing	Pain Catastrophizing Scale	0, 3 months	I: 18.6 (9.5)<br>C: 19.1 (9.6)	I: 13.5 (11.3)<br>C: 17.8 (11.0)	Yes
Van der Weegen, 2015	General self-efficacy	General Self-Efficacy Scale	0, post-intervention	I: 3.2 (0.5)<br>C: 3.1 (0.5)	I: 3.3 (0.4)<br>C: 3.2 (0.4)	No
	Exercise self-efficacy	Exercise Self-Efficacy Scale	0, post-intervention	I: 55.4 (17.0)<br>C: 54.0 (19.2)	I: 59.7 (17.3)<br>C: 54.5 (17.4)	No
**Dealing with the chronic condition: behavioral measures**						
Buhrman, 2004	Coping strategy: Increase activity level	Coping Strategy Questionnaire: subscale increase activity level	0, 8 weeks	I: 14.4 (5.0)<br>C: 17.3 (6.1)	I: 14.8 (5.6)<br>C: 16.9 (6.3)	No
	Pain interference with daily activities	Multidimensional Pain Inventory: subscale pain interference	0, 8 weeks	I: 3.6 (1.2)<br>C: 3.9 (1.3)	I: 3.2 (1.4)<br>C: 3.5 (1.2)	No
Buhrman, 2011	Coping strategy:	Coping Strategy Questionnaire:	0, 12 weeks	I: 16.0 (6.0)<br>C: 15.6 (4.5)	I: 14.3 (5.4)<br>C: 15.9 (5.7)	No

	Increase activity level	subscale increase activity level				
	Pain interference with daily activities	Multidimensional Pain Inventory: subscale pain interference	0, 12 weeks	I: 3.6 (1.2)<br>C:3.9 (1.3)	I: 3.2 (1.4)<br>C: 3.5 (1.2)	No
Buhrman, 2013	Coping Strategy: Increase activity level	Coping Strategy Questionnaire: subscale increase activity level	0, 8 weeks	I: 16.32 (6.41)<br>C: 16.05 (5.15)	I: 15.76 (5.56)<br>C: 18.07 (4.60)	No
	Coping Strategy: Pain behavior	Coping Strategy Questionnaire: subscale pain behavior	0, 8 weeks	I: 18.26 (4.85)<br>C: 18.71 (4.67)	I: 18.07 (4.60)<br>C: 18.69 (5.39)	No
	Coping Strategy: the extent to which a person follows their activities regardless of pain	Chronic pain acceptance questionnaire: subscale activities engagement	0, 8 weeks	I: 22.84 (11.02)<br>C: 21.18 (9.70)	I: 28.62 (11.15)<br>C: 22.22 (11.17)	Yes
	Coping Strategy: the extent to which a person believes that avoiding activities prevents pain	Chronic pain acceptance questionnaire: subscale pain willingness	0, 8 weeks	I: 18.13 (8.85)<br>C: 20.61 (8.68)	I: 23.53 (8.32)<br>C: 21.53 (7.94)	Yes
	Pain interference with daily activities	Multidimensional Pain Inventory: subscale pain interference	0, 8 weeks	I: 4.76 (0.88)<br>C: 4.85 (0.89)	I: 4.37 (1.09)<br>C:4.94 (0.93)	Yes
Buhrman, 2015	Coping Strategy: Increase activity level	Coping Strategy Questionnaire: subscale increase activity level	0, 8 weeks	I: 14.93 (5.13)<br>C: 15.92 (5.23)	I: 16.40 (5.69)<br>C: 15.76 (5.07)	No
	Coping Strategy: Pain behavior	Coping Strategy Questionnaire: subscale pain behavior	0, 8 weeks	I: 17.07 (5.00)<br>C: 17.67 (4.14)	I: 17.64 (5.38)<br>C:18.33 (5.50)	No
	Coping Strategy: the extent to which a person follows their activities regardless of	Chronic pain acceptance questionnaire: subscale activities engagement	0, 8 weeks	I: 38.00 (11.45)<br>C: 38.54 (11.68)	I: 45.11 (12.03)<br>C: 40.37 (9.16)	Yes

	pan					
	Coping Strategy: the extent to which a person believes that avoiding activities prevents pain	Chronic pain acceptance questionnaire: subscale pain willingness	0, 8 weeks	I: 30.46 (8.00)<br>C: 31.58 (7.76)	I: 34.73 (7.15)<br>C: 32.38 (5.72)	No
	Pain interference with daily activities	Multidimensional Pain Inventory: subscale pain interference		I: 3.91 (1.20)<br>C: 4.14 (1.03)	I: 3.31 (1.29)<br>C: 3.80 (1.21)	No
Ferwerda, 2017	Compliance to standard rheumatological care	5-point Likert scales for rheumatologic care	0, post-intervention	I: 4.15 (0.92)<br>C: 4.20 (0.89)	I: 4.43 (0.67)<br>C: 4.28 (0.71	No
Friessen, 2017	Pain interference with daily activities	Brief Pain Inventory: subscale interference	0, 8 weeks	I: 6.56 (1.90)<br>C: 7.48 (1.71)	I: 5.46 (2.11)<br>C: 7.32 (1.58)	No
Van der Meer, 2009	Adequacy of asthma control	Asthma Control Questionnaire	0, 12 months	I: 1.12 (NM)<br>C:1.11 (NM)	I: 0.59 (NM)<br>C: 1.04 (NM)	Yes
Torbjørnsen, 2014	Self-management: skills and technique acquisition	Hei-Q	0, 4 months	I: 2.87 (0.43)<br>C: 2.92 (0.34)	I: 3.04 (0.52)<br>C: 2.92 (0.56)	Yes
	Self-management: Health service navigation	Hei-Q	0, 4 months	I: 3.08 (0.43)<br>C: 3.13 (0.40)	I: 3.27 (0.56)<br>C: 3.20 (0.54)	Yes
Trompetter, 2015	Awareness of personal values and the degree to which this guide person's actions in daily life	Engaged Living Scale	0, 3 months	I: 51.5 (10.4)<br>C: 49.8 (9.1)	I: 55.1 (12.4)<br>C: 52.8 (11.8)	No
	Pain interference with daily activities	Multidimensional Pain Inventory: subscale pain interference	0, 3 months	I: 32.3 (9.8)<br>C: 33.3 (9.8)	I: 28.7 (12.0)<br>C: 32.1 (11.5)	No
**Emotional outcomes**						
Buhrman, 2004	Anxiety	Hospital Anxiety and Depression Scale	0, 8 weeks	I: 7.4 (4.5)<br>C: 7.0 (3.3)	I: 7.2 (4.0)<br>C: 6.0 (3.3)	No

	Depression	Hospital Anxiety and Depression Scale	0, 8 weeks	I: 6.9 (4.8)<br>C: 6.6 (4.1)	I: 6.0 (4.7)<br>C: 5.4 (4.0)	No
	Affective distress	Multidimensional Pain Inventory: subscale affective distress	0, 8 weeks	I: 2.9 (0.9)<br>C: 3.0 (0.6)	I: 2.8 (0.9)<br>C: 3.1 (0.6)	No
Buhrman, 2011	Anxiety	Hospital Anxiety and Depression Scale	0, 12 weeks	I: 7.6 (3.7)<br>C: 7.6 (5.1)	I: 5.8 (3.5)<br>C: 7.0 (6.0)	No
	Depression	Hospital Anxiety and Depression Scale	0, 12 weeks	I: 6.3 (4.2)<br>C: 6.3 (4.5)	I: 4.9 (3.6)<br>C: 6.3 (5.2)	No
	Affective distress	Multidimensional Pain Inventory: subscale affective distress	0, 12 weeks	I: 2.9 (0.9)<br>C: 3.0 (0.6)	I: 2.8 (0.9)<br>C: 3.1 (0.6)	No
Buhrman, 2013	Anxiety	Hospital Anxiety and Depression Scale	0, 8 weeks	I: 9.89 (4.19)<br>C: 9.13 (4.26)	I: 8.97 (4.33)<br>C: 9.67 (3.50)	Yes
	Depression	Hospital Anxiety and Depression Scale	0, 8 weeks	I: 9.58 (4.57)<br>C: 9.63 (4.04)	I: 8.85 (4.40)<br>C: 10.52 (3.77)	Yes
	Affective distress	Multidimensional Pain Inventory: subscale affective distress	0, 8 weeks	I: 3.32 (0.78)<br>C: 3.14 (0.73)	I: 3.08 (0.74)<br>C: 3.31 (0.64)	Yes
Buhrman, 2015	Anxiety	Beck Anxiety Inventory	0, 8 weeks	I: 20.36 (9.54)<br>C:17.83 (7.56)	I: 11.99 (8.13)<br>C: 14.57 (6.81)	Yes
	Depression	Montgomery Asberg Depression Rating Scale	0, 8 weeks	I: 23.14 (6.94)<br>C: 20.83 (6.19)	I: 15.77 (7.79)<br>C: 17.95 (6.51)	Yes
	Affective distress	Multidimensional Pain Inventory: subscale affective distress	0, 8 weeks	I: 3.25 (0.61)<br>C: 3.26 (0.81)	I: 3.17 (0.56)<br>C:3.31 (0.64)	No
Dear, 2015 *regular contact group	Depression	Patient Health Questionnaire 9-item	0, 8 weeks	I: 11.55 (5.88)<br>C: 10.37 (5.47)	I: 6.30 (4.57)<br>C: 11.11 (5.51)	Yes
	Anxiety	Generalized Anxiety Disorder 7-item	0, 8 weeks	I: 8.40 (5.52)<br>C: 8.21 (5.92)	I: 4.91 (4.40)<br>C: 7.89 (5.29)	Yes
Dear, 2015 *optional contact group	Depression	Patient Health Questionnaire 9-item	0, 8 weeks	I: 10.60 (5.33)<br>C: 10.37 (5.47)	I: 7.20 (5.25)<br>C: 11.11 (5.51)	Yes
	Anxiety	Generalized Anxiety Disorder 7-item	0, 8 weeks	I: 7.98 (4.67)<br>C: 8.21 (5.92)	I: 5.66 (4.94)<br>C: 7.89 (5.29)	Yes
Ferwerda, 2016	Depression	Beck Depression Inventory	0, post-intervention	I: 11.53 (6.99)<br>C: 13.38 (6.46)	I: 8.16 (5.67)<br>C: 12.27 (5.97)	Yes

	Negative mood	Impact of Rheumatic Diseases on General Health and Lifestyle	0, post-intervention	I: 4.07 (2.56)<br>C: 5.42 (4.21)	I: 3.25 (2.39)<br>C: 4.95 (4.17)	Yes
	Anxiety	Impact of Rheumatic Diseases on General Health and Lifestyle	0, post-intervention	I: 20.82 (4.85)<br>C: 21.40 (4.85)	I: 18.12 (4.13)<br>C: 20.61 (4.99)	Yes
Friessen, 2017	Anxiety	Generalized Anxiety Disorder 7-item (GAD7)	0, 8 weeks	I: 10.87 (4.65)<br>C: 9.93 (4.88)	I: 7.83 (5.70)<br>C: 9.98 (5.15)	Yes
	Depression	Patient Health Questionnaire 9-item(PHQ9)	0, 8 weeks	I: 14.07 (4.66)<br>C: 14.07 (4.93)	I: 10.13 (5.30)<br>C: 14.00 (5.44)	Yes
	Anxiety	Hospital Anxiety and Depression Scale	0, 8 weeks	I: 11.60 (4.00)<br>C: 10.17 (3.98)	I: 9.22 (4.33)<br>C: 10.43 (4.69)	Yes
	Depression	Hospital Anxiety and Depression Scale	0, 8 weeks	I: 9.90 (3.37)<br>C: 9.97 (3.82)	I: 7.97 (3.55)<br>C: 10.17 (3.42)	Yes
Hunt, 2009	Anxiety	Anxiety Sensitivity Index	0, 5 weeks	I: 2.9 (0.91)<br>C: 2.7 (0.99)	I: 1.9 (0.93)<br>C: 2.5 (0.95)	Yes
Jasper, 2014	Tinnitus associated distress	Mini-Tinnitus Questionnaire	0, 10 weeks	I: 12.20 (4.58)<br>C: 12.50 (4.83)	I: 7.44 (5.30)<br>C: 11.09 (5.77)	Yes
	Tinnitus associated distress	Tinnitus Handicap Inventory	0, 10 weeks	I: 40.34 (17.64)<br>C: 40.23 (20.54)	I: 26.67 (20.75)<br>C: 37.46 (18.94)	Yes
	Anxiety	Hospital Anxiety and Depression Scale	0, 10 weeks	I: 7.41 (3.56)<br>C: 8.00 (4.24)	I: 5.44 (3.23)<br>C: 7.67 (4.68)	Yes
	Depression	Hospital Anxiety and Depression Scale	0, 10 weeks	I: 5.95 (4.21)<br>C: 6.43 (4.48)	I: 4.41 (3.72)<br>C: 5.88 (4.41)	No
Moss Morris, 2012	Anxiety	Hospital Anxiety and Depression Scale	0, 10 weeks	I: 8.26 (4.31)<br>C: 9.56 (4.50)	I: 6.44 (3.91)<br>C: 11.65 (5.26)	Yes
	Depression	Hospital Anxiety and Depression Scale	0, 10 weeks	I: 7.96 (3.64)<br>C: 6.75 (2.72)	I: 5.18 (3.38)<br>C: 8.73 (3.62)	Yes
Steel, 2016	Depression	Center for Epidemiologic Studies Depression Scale	0, 6 months	I: 25.9 (9.8)<br>C: 25.49 (6.9)	I: 15.3 (10.5)<br>C: 24.7 (15.1)	No
Trompetter, 2015	Anxiety	Hospital Anxiety and Depression Scale	0, 3 months	I: 7.2 (3.3)<br>C: 6.9 (3.3)	I: 6.0 (3.8)<br>C: 6.1 (3.6)	No

	Depression	Hospital Anxiety and Depression Scale	0, 3 months	I: 6.1 (3.4)<br>C: 6.1 (3.4)	I: 5.1 (3.7)<br>C: 5.8 (3.5)	No
	Positive mental health	Mental Health Continuum-Short Form	0, 3 months	I: 52.6 (11.8)<br>C:49.9 (12.8)	I: 54.7 (12.2)<br>C: 52.6 (14.3)	No
Van Beugen, 2016	Negative mood	Impact of chronic Skin Disease on Daily Life	0, post-treatment	I: 5.29 (3.77)<br>C: 5.39 (3.72)	I: 3.69 (3.36)<br>C: 4.60 (3.21)	No
	Anxiety	Impact of chronic Skin Disease on Daily Life	0, post-treatment	I: 21.85 (4.61)<br>C: 22.10 (4.58)	I: 19.36 (4.37)<br>C: 21.02 (5.74)	No
	Depression	Beck Depression Inventory	0, post-treatment	I: 12.78 (7.50)<br>C: 11.50 (6.23)	I: 8.46 (5.34)<br>C: 8.89 (6.82)	No
**Quality of life**						
Buhrman, 2011	Generic quality of life	Quality Of Life Inventory	0, 12 weeks	I: 1.2 (1.4)<br>C: 1.8 (1.5)	I: 1.7 (1.4)<br>C: 1.1 (1.6)	Yes
Buhrman, 2013	Generic quality of life	Quality Of Life Inventory	0, 8 weeks	I: 0.26 (2.18)<br>C: 0.15 (1.97)	I: 0.56 (2.07)<br>C: 0.39 (1.77)	No
Buhrman, 2015	Generic quality of life	Quality of Life Inventory	0, 8 weeks	I: 0.65 (1.65)<br>C: 0.70 (1.39)	I: 1.38 (1.78)<br>C: 1.39 (1.59)	No
Dlugonski, 2012	Multiple Sclerosis-specific health related quality of life: physical impact	Multiple Sclerosis Impact Scale	0, 12 weeks	I: 44.3 (19.8)<br>C: 38.3 (19.0)	I: 44.8 (21.2)<br>C: 41.1 (20.6)	No
	Multiple Sclerosis-specific health related quality of life: psychological impact	Multiple Sclerosis Impact Scale	0, 12 weeks	I: 20.0 (8.2)<br>C: 18.5 (8.0)	I: 19.5 (7.9)<br>C: 19.6 (9.2)	No
Ferwerda, 2017	Health related quality of life: Role impairment (physical)	RAND-36 subscale Role impairment (physical)	0, post-intervention	I: 39.58 (39.94)<br>C: 26.49 (38.65)	I: 48.91 (45.02)<br>C: 33.19 (39.84)	No
	Health related quality of life: Role impairment (emotional)	RAND-36 subscale Role impairment (emotional)	0, post-intervention	I: 69.44 (39.91)<br>C: 58.82 (46.47)	I: 83.33 (28.76)<br>C: 62.15 (45.26)	Yes
Friessen, 2017	Quality of life, physical component	Medical Outcomes Study Short Form (SF 12)	0, 8 weeks	I: 30.81 (7.82)<br>C: 32.17 (7.35)	I: 34.70 (7.94)<br>C: 32.82 (8.20)	Yes

	Quality of life, mental component	Medical Outcomes Study Short Form (SF 12)	0, 8 weeks	I: 34.42 (8.52)<br>C: 36.12 (7.60)	I: 39.62 (11.22)<br>C: 38.95 9.16)	No
Hunt, 2009	Irritable bowel-specific health related quality of life	Irritable Bowel Syndrome Quality of Life Impairment	0, 5 weeks	I: 122 (27)<br>C: 84 (26)	I: 123 (26)<br>C: 111 (25)	Yes
Ljotsson, 2011	Irritable bowel-specific health related quality of life	Irritable Bowel Syndrom Quality of Life Instrument	0, 10 weeks	I: 67.4 (20.9)<br>C: 76.1 (18.8)	I: 82.6 (13.4)<br>C: 67.4 (23.1)	Yes
Van Beugen, 2016	Health related quality of life: Role impairment (physical)	RAND-36 subscale Role impairment (physical)	0, post-intervention	I: 53.02 (41.90)<br>C: 58.89 (43.09)	I: 72.09 (36.68)<br>C:73.94 (40.03)	Yes
	Health related quality of life: Role impairment (emotional)	RAND-36 subscale Role impairment (emotional)	0, post-intervention	I: 78.16 (35.07)<br>C: 67.54 (39.89)	I: 86.6 (23.09)<br>C: 76.09 (38.27)	No
Van der Meer 2009	Asthma-specific health related quality of life	Asthma Quality of Life Questionnaire	0, 12 months	I: 5.73 (NM)<br>C: 5.79 (NM)	I: 6.29 (NM)<br>C: 5.97 (NM)	Yes
Van der Weegen, 2015	Health related quality of life: Role impairment (physical)	RAND-36 subscale Role impairment (physical)	0, post-intervention	I: 42.5 (11.1)<br>C: 45.8 (9.4)	I: 45.2 (9.5)<br>C: 47.0 (10.0)	No
	Health related quality of life: Role impairment (emotional)	RAND-36 subscale Role impairment (emotional)	0, post-intervention	I: 48.2 (10.3)<br>C: 50.1 (9.5)	I: 48.8 (10.6)<br>C: 47.7 (9.8)	Yes
Steel, 2016	Cancer-specific health related quality of life	Functional Assessment of Cancer Therapy-General	0, 6 months	I: 67.2 (17.1)<br>C: 65.8 (16.9)	I: 82.4 (15.2)<br>C: 63.2 (21.5)	Yes
