# Supplementary material for: Blended Interventions to Change Behavior in Patients With Chronic Somatic Disorders: Systematic Review
Source: J Med Internet Res. 2017 Dec 21;19(12):e418. doi: 10.2196/jmir.8108 (PMC5754569; doi:10.2196/jmir.8108)
Supplement: Multimedia Appendix 5 [file jmir_v19i12e418_app5.pdf]

| Author, year of publication | Outcome measure | Instrument | Time of measurement (pre- and post-treatment) | Pre-treatment mean (SD) | Post-treatment mean (SD) | Significant effect |
|---|---|---|---|---|---|---|
| **Symptoms and signs** | | | | | | |
| Allen, 2013 *intensive counseling | Body weight | Weighing scale | 0, 6 months | I: 100.3 (16.5)<br>C:96.4 (16.9) | I: 94.9 (4.0)<br>C:94.6 (3.7) | No |
| | BMI | Scale and meter | 0, 6 months | I: 34.3 (3.9)<br>C: 35.3 (4.1) | I: 32.5 (1.3)<br>C: 34.7 (1.3) | No |
| | Male waist circumference | Measuring tape | 0, 6 months | I: 119.4 (11.6)<br>C: 113.8 (23.0) | I: 112.4 (2.6)<br>C: 110.4 (8.3) | No |
| | Female waist circumference | Measuring tape | 0, 6 months | I: 109.7 (11.4)<br>C: 105.5 (11.1) | I: 104.0 (3.7)<br>C: 110.4 (8.3) | No |
| Allen, 2013 *less intensive counseling | Body weight | Weighing scale | 0, 6 months | I: 96.8 (14.8)<br>C: 96.4 (16.9) | I: 93.5 (5.9)<br>C: 94.6 (3.7) | No |
| | BMI | Scale and meter | 0, 6 months | I: 33.5 (3.5)<br>C: 35.3 (4.1) | I: 32.4 (2.0)<br>C: 34.7 (1.3) | No |
| | Male waist circumference | Measuring tape | 0, 6 months | I: 116.4 (4.6)<br>C: 113.8 (23.0) | I: 109.9 (0.35)<br>C: 110.4 (8.3) | No |
| | Female waist circumference | Measuring tape | 0, 6 months | I: 108.7 (8.4)<br>C: 105.5 (11.1) | I: 105.1 (7.9)<br>C: 110.4 (8.3) | No |
| Dear, 2015 *regular contact group | Average pain | Wilcinson Brief Pain Questionnaire | 0, 8 weeks | I: 5.74 (1.72)<br>C: 5.72 (1.63) | I: 4.86 (1.79)<br>C: 5.20 (1.80) | No |
| Dear, 2015 *optional contact group | Average pain | Wilcinson Brief Pain Questionnaire | 0, 8 weeks | I: 5.54 (1.74)<br>C: 5.72 (1.63) | I: 4.85 (1.73)<br>C: 5.20 (1.80) | No |
| Glasgow, 2010 | BMI | Scale and meter | 0, 4 months | I: 35.2 (6.78)<br>C: 34.5 (6.28) | I: 35.1 (6.83)<br>C: 34.4 (6.27) | Yes |
| | Hemoglobin | Bio-Rad Variant II Turbo liquid by high pressure liquid chromatography | 0, 4 months | I: 8.26 (1.75)<br>C: 8.01 (1.85) | I: 8.05 (1.48)<br>C: 7.84 (1.67) | No |
| | Lipid ratio (total / HDL) | Modular chemistry analyzer, Abell Kendall method | 0, 4 months | I: 4.04 (1.11)<br>C: 4.00 (1.25) | I: 3.94 (1.16)<br>C: 3.84 (1.06) | No |

| | | | | | | |
|---|---|---|---|---|---|---|
| | Mean arterial blood pressure | Blood pressure meter | 0, 4 months | I: 95.12 (10.54)<br>C: 95.42 (10.40) | I: 94.58 (10.50)<br>C: 94.27 (10.20) | No |
| Torbjørnsen, 2014 | Hemoglobin | Blood test | 0, 4 months | I: 8.2 (1.08)<br>C: 8.1 (1.09) | I: 7.8 (1.44)<br>C: 7.8 (0.91) | No |
| Trompetter, 2015 | Pain | Pain NRS | 0, 3 months | I: 6.3 (1.6)<br>C: 6.1 (1.6) | I: 5.4 (2.2)<br>C: 5.9 (2.3) | Yes |
| Van den Berg, 2006 | Disease activity: pain, swelling and tenderness | Reumatoid Arthritis Disease Activity: DAS28 | 0, 12 months | I: 3.5 (2.3)<br>C: 3.3 (2.1) | I: 3.1 (1.6)<br>C: 2.8 (2.0) | No |
| Yardley, 2014<br>*basic nurse support | Body weight | Weighing scale | 0, 6 months | I: 103.43 (25.23)<br>C: 98.25 (18.11) | I: 97.92 (5.80)<br>C: 99.00 (5.75) | No |
| Yardley, 2014<br>*regular nurse support | Body weight | Weighing scale | 0, 6 months | I: 100.36 (19.38)<br>C: 98.25 (18.11) | I: 97.14 (5.74)<br>C: 99.00 (5.75) | No |
| **Daily activity related limitations** | | | | | | |
| Allen, 2013<br>* intensive counseling | Self-reported activity | Stanford 7-Day Physical Activity Recall | 0, 6 months | I:4.9 (5.7)<br>C:3.5 (3.7) | I: 2.9 (5.4)<br>C:3.3 (5.1) | No |
| Allen, 2013<br>*less intensive counseling | Self-reported activity | Stanford 7-Day Physical Activity Recall | 0, 6 months | I: 5.3 (5.4)<br>C:3.5 (3.7) | I:1.7 (5.5)<br>C:3.3 (5.1) | No |
| Van den Berg, 2006 | Functional ability | MACTAR | 0, 12 months | I: 51.0 (4.0)<br>C: 50.0 (4.0) | I: 55.2 (8.1)<br>C: 52.5 (10.1) | Yes |
| | Rheumatoid related disabilities | Health Assessment Questionnaire | 0, 12 months | I: 0.75 (1.13)<br>C: 0.75 (0.75) | I: 0.66 (0.35)<br>C: 0.71 (0.16) | Yes |
| Dear, 2015<br>*regular contact group | Backpain associated disability in daily activities | Roland-Morris Disability Questionnaire | 0, 8 weeks | I: 13.47 (5.23)<br>C: 13.92 (5.06) | I: 11.05 (5.63)<br>C: 11.36 (5.22) | No |
| Dear, 2015<br>*optional | Backpain associated | Roland-Morris Disability | 0, 8 weeks | I: 13.24 (5.60)<br>C: 13.92 (5.06) | I: 10.95 (.84)<br>C: 11.36 (5.22) | No |

| | | | | | | |
|---|---|---|---|---|---|---|
| contact group | disability in daily activities | Questionnaire | | | | |
| Glasgow, 2010 | Physical activity | CHAMPS instrument | 0, 4 months | I: 3664 (2959)<br>C: 4294 (3054) | I: 3697 (3272)<br>C: 4146 (3578) | No |
| Liebreich, 2009 | Leisure-time Physical activity | Modified version Godin Leisure-Time Exercise Questionnaire | 0, 3 months | I: 483 (620)<br>C: 501 (582) | I: 654 (659)<br>C: 490 (562) | Yes |
| McKay, 2001 | Moderate-to-vigorous exercise | Behavioral Risk Factor Surveillance system | 0, 2 months | I: 5.6 (6.2)<br>C: 7.3 (6.2) | I : 17.6 (15.3)<br>C : 18.0 (17.3) | No |
| | Walking | Behavioral Risk Factor Surveillance system | 0, 2 months | I: 6.4 (6.2)<br>C: 8.4 (8.4) | I: 12.5 (9.5)<br>C: 16.8 (22.8) | No |
| Van den Berg, 2006 | Meeting physical activity recommendations | Self-reported, percentage of total sample | 0, 12 months | I: 0 (0)<br>C: 0 (0) | I: 19 (26)<br>C: 11 (15) | No |
| | Physical activity score | Number of acceleration per 5-minute period throughout the day, Actilog V3.0 activity monitor | 0, 12 months | I: 72.0 (33.5)<br>C: 79.0 (25.8) | I: 72.3 (34.0)<br>C: 80.7 (30.6) | No |
| Trompetter, 2015 | Disruption of daily activities by chronic pain | Pain Disability Index | 0, 3 months | I: 36.0 (12.7)<br>C: 36.4 (12.7) | I: 30.6 (14.5)<br>C: 34.6 (14.3) | No |
| **Dealing with the chronic condition: cognitive measures** | | | | | | |
| Dear, 2015<br>*regular contact group | Pain Self-efficacy | Pain Self-efficacy Questionnaire | 0, 8 weeks | I: 28.86 (12.93)<br>C: 26.80 (11.52) | I: 35.94 (12.98)<br>C: 33.21 (11.97) | Yes |
| | Fear of movement | TAMPA Scale of Kinesiophobia | 0, 8 weeks | I: 38.59 (7.99)<br>C: 39.35 (7.37) | I: 34.48 (7.00)<br>C: 34.59 (6.80) | No |
| | Pain acceptance | Chronic Pain Acceptance | 0, 8 weeks | I: 22.58 (7.53)<br>C: 22.26 (7.19) | I: 26.79 (6.69)<br>C: 25.76 (6.65) | No |

| | | | | | | |
|---|---|---|---|---|---|---|
| | | Questionnaire | | | | |
| Dear, 2015 *optional contact group | Pain Self-efficacy | Pain Self-efficacy Questionnaire | 0, 8 weeks | I: 28.61 (14.08) C: 26.80 (11.52) | I: 33.60 (13.83) C: 33.21 (11.97) | No |
| | Fear of movement | TAMPA Scale of Kinesiophobia | 0, 8 weeks | I: 37.60 (8.40) C: 39.35 (7.37) | I: 34.88 (7.80) C: 34.59 (6.80) | No |
| | Pain acceptance | Chronic Pain Acceptance Questionnaire | 0, 8 weeks | I: 23.30 (7.72) C: 22.26 (7.19) | I: 25.40 (8.01) C: 25.76 (6.65) | No |
| Liebreich, 2009 | Self-efficacy in performing regular physical activity | Self-efficacy 12-item scale, Plotnikoff et al. | 0, 3 months | I: 3.00 (0.74) C: 3.01 (0.71) | I: 2.99 (0.84) C: 2.82 (0.84) | No |
| | Outcome expectations of engaging in physical activity | Modified version of the Decisional Balance Scale and Physical Activity Expectations Scale | 0, 3 months | I: 4.66 (0.87) C: 4.62 (0.37) | I: 4.57 (0.54) C: 4.57 (0.42) | No |
| | The perceived importance of outcome expectations | Perceived importance of the Outcome Expectations | 0, 3 months | I: 2.73 (0.29) C: 2.71 (0.34) | I: 2.69 (0.39) C: 2.65 (0.36) | No |
| | The degree to which participants rewarded themselves ans set realistic goals | Reinforcement 4-item scale, Marcus et al. | 0, 3 months | I: 2.73 (0.66) C: 2.58 (0.84) | I: 2.83 (0.74) C: 2.70 (0.91) | No |
| | Emotional coping response | Emotional well-being subscale | 0, 3 months | I: 2.93 (0.76) C: 2.78 (0.67) | I: 2.90 (0.67) C: 2.90 (0.67) | No |
| | Participants'indication of how true a variety | Subscale of Behavior Regulation in | 0, 3 months | I: 3.81 (0.54) C: 3.94 (0.53) | I: 3.56 (0.91) C: 3.75 (1.05) | No |

| | | | | | | |
|---|---|---|---|---|---|---|
| | of reasons to exercise were (e.g. 'I value the benefits of exercise') | Exercise Questionnaire | | | | |
| | Experienced confidence in performing physical activity | Behavioral capacity 4-item scale, Rogers et al. | 0, 3 months | I: 3.10 (1.18)<br>C: 3.25 (0.98) | I: 3.23 (1.32)<br>C: 2.97 (0.98) | Yes |
| | Indication of how often external situations prevented participants from getting a physical active lifestyle | Situation 17-item scale, Rogers et al. | 0, 3 months | I: 2.41 (0.66)<br>C: 2.11 (0.55) | I: 2.35 (0.67)<br>C: 2.16 (0.59) | No |
| | Experienced social support in getting a physical active lifestyle | Social support 2-item scale, Cournya et al. | 0, 3 months | I: 5.06 (1.52)<br>C: 4.58 (1.99) | I: 4.18 (2.10)<br>C: 4.27 (2.05) | No |
| | The degree to which participants observed others being physical active | Observational learning 2-item scale, Plotnikoff et al. | 0, 3 months | I: 4.14 (0.78)<br>C: 3.25 (0.98) | I: 3.96 (0.92)<br>C: 2.97 (0.98) | No |
| Trompetter, 2015 | Positive mental health | Mental Health Continuum-Short Form | 0, 3 months | I: 52.6 (11.8)<br>C: 53.1 (11.8) | I: 54.7 (12.2)<br>C: 55.9 (15.2) | No |
| | Avoidance of pain and cognitive fusion with | Psychological Inflexibility in Pain Scale | 0, 3 months | I: 55.0 (11.6)<br>C:55.1 (11.6) | I: 40.7 (13.8)<br>C: 46.3 (14.1) | Yes |

| | | | | | | |
|---|---|---|---|---|---|---|
| | pain | | | | | |
| | Mindfulness skills | Five Facet Mindfulness Questionnaire-Short Form | 0, 3 months | I:81.4 (10.7)<br>C:83.2 (10.7) | I:86.7 (12.2)<br>C: 88.8 (12.5) | No |
| | Pain catastrophizing | Pain Catastrophizing Scale | 0, 3 months | I: 18.6 (9.5)<br>C: 17.6 (10.2) | I: 13.5 (11.3)<br>C: 15.6 (11.7) | No |
| **Dealing with the chronic condition: behavioral measures** | | | | | | |
| Nobis, 2015 | Coping with diabetes | Acceptance and Action Diabetes Questionnaire | 0, 2 months | I: 36.93 (8.96)<br>C: 36.56 (10.27) | I: 34.13 (8.45)<br>C: 36.11 (9.86) | Yes |
| | Diabetes specific self-management | Diabetes Self-Management Questionnaire | 0, 2 months | I: 4.78 (0.65)<br>C:4.68 (0.65) | I: 4.76 (0.55)<br>C: 4.72 (0.62) | No |
| Torbjørnsen, 2014 | Self-management: skills and technique acquisition | Hei-Q | 0, 4 months | I: 2.87 (0.43)<br>C: 2.95 (0.46) | I: 3.04 (0.52)<br>C: 2.98 (0.62) | No |
| | Self-management: Health service navigation | Hei-Q | 0, 4 months | I: 3.08 (0.43)<br>C: 3.14 (0.51) | I: 3.27 (0.56)<br>C: 3.21 (0.60) | Yes |
| Trompetter, 2015 | Awareness of personal values and the degree to which this guide person's actions in daily life | Engaged Living Scale | 0, 3 months | I:51.5 (10.4)<br>C: 51.5 (9.9) | I: 55.1 (12.4)<br>C: 56.1 (12.8) | No |
| | The degree to which pain interferes with daily activities | Multidimensional Pain Inventory | 0, 3 months | I: 32.3 (9.8)<br>C: 32.2 (9.8) | I: 28.7 (12.0)<br>C: 32.7 (12.3) | Yes |
| **Emotional outcomes** | | | | | | |
| Dear, 2015<br>*regular | Depression | Patient Health Questionnaire | 0, 8 weeks | I: 11.55 (5.88)<br>C: 10.90 (4.76) | I: 6.30 (4.57)<br>C: 6.96 (4.29) | No |

| | | | | | | |
|---|---|---|---|---|---|---|
| contact group | | 9-item | | | | |
| | Anxiety | Generalized Anxiety Disorder 7-item | | I: 8.40 (5.52)<br>C: 8.28 (4.60) | I: 4.91 (4.40)<br>C: 5.16 (3.91) | No |
| Dear, 2015<br>*optional contact group | Depression | Patient Health Questionnaire 9-item | 0, 8 weeks | I: 10.60 (5.33)<br>C: 10.90 (4.76) | I: 7.20 (5.25)<br>C: 6.96 (4.29) | No |
| | Anxiety | Generalized Anxiety Disorder 7-item | | I: 7.98 (4.67)<br>C: 8.28 (4.60) | I: 5.66 (4.94)<br>C: 5.16 (3.91) | No |
| McKay, 2001 | Depression | 10-item version of the Center for Epidemiologic Studies Depression scale | 0, 2 months | I: 16.9 (11.6)<br>C: 17.6 (10.4) | I: 14.9 (12.5)<br>C: 19.9 (14.2) | No |
| Nobis, 2015 | Depressive symptom severity | Center for Epidemiologic Studies Depression Scale | 0, 2 months | I: 32.7 (6.95)<br>C:32.53 (7.51) | I: 21.08 (8.84)<br>C: 28.90 (8.65) | Yes |
| | Depressive symptoms | Hospital Anxiety and Depression Scale | 0, 2 months | I: 11.99 (3.23)<br>C: 11.69 (3.08) | I: 8.12 (3.92)<br>C: 11.26 (3.72) | Yes |
| | Emotional distress related to living with DM | Problem Areas in Diabetes Scale | 0, 2 months | I: 10.24 (4.27)<br>C: 10.57 (4.52) | I: 8.35 (3.94)<br>C: 10.87 (4.67) | Yes |
| Trompetter, 2015 | Anxiety | Hospital Anxiety and Depression Scale | 0, 3 months | I: 7.2 (3.3)<br>C: 6.5 (3.4) | I: 6.0 (3.8)<br>C: 5.7 (3.8) | No |
| | Depression | Hospital Anxiety and Depression Scale | 0, 3 months | I: 6.1 (3.4)<br>C: 7.5 (3.3) | I: 5.1 (3.7)<br>C: 5.9 (3.9) | No |

| Quality of life | | | | | | |
|---|---|---|---|---|---|---|
| De Boer, 2014 | Health related quality of life: Physical functioning | RAND-36 subscale Physical functioning | 0, 4 months | I: 50.10 (17.20) C: 52.50 (22.94) | I: 58.50 (22.37) C: 55.88 (22.35) | Yes |
| | Health related quality of life: Social functioning | RAND-36 subscale Social functioning | 0, 4 months | I: 49.34 (23.74) C: 47.92 (23.79) | I: 65.79 (26.95) C: 54.17 (27.00) | Yes |
| | Health related quality of life: Role impairment (physical) | RAND-36 subscale Role impairment (physical) | 0, 4 months | I: 15.79 (29.12) C: 15.63 (24.24) | I: 37.24 (41.89) C: 22.19 (34.89) | No |
| | Health related quality of life: Role impairment (emotional) | RAND-36 subscale Role impairment (emotional) | 0, 4 months | I: 59.65 (42.42) C: 57.97 (44.06) | I: 71.93 (41.96) C: 67.39 (40.97) | No |
| | Health related quality of life: Mental health | RAND-36 subscale Mental health | 0, 4 months | I: 62.74 (18.95) C: 65.83 (18.80) | I: 70.11 (20.76) C: 66.17 (17.61) | No |
| | Health related quality of life: Vitality | RAND-36 subscale Vitality | 0, 4 months | I: 41.32 (15.35) C: 39.79 (16.84) | I: 52.63 (20.30) C: 40.63 (14.69) | Yes |
| | Health related quality of life: Pain | RAND-36 subscale Pain | 0, 4 months | I: 37.16 (14.73) C: 38.76 (17.87) | I: 50.70 (18.60) C:39.80 (19.35) | Yes |
| | Health related quality of life: General health appraisal | RAND-36 subscale General health appraisal | 0, 4 months | I: 49.33 (23.35) C: 42.50 (22.84) | I: 56.63 (27.69) C: 43.54 (20.51) | No |
| | Health related quality of life: Perceived health change | RAND-36 subscale Perceived health change | 0, 4 months | I: 28.75 (24.70) C: 38.54 (30.38) | I: 51.25 (23.61) C: 38.54 (34.56) | Yes |
| Van den Berg, 2006 | Disease related quality of life | Rheumatoid Arthritis | 0, 12 months | I: 10.0 (10.2) C: 10.0 (9.5) | I: 8.7 (6.1) C: 9.4 (3.6) | Yes |

| | | | | | | |
|---|---|---|---|---|---|---|
| | | Quality of Life | | | | |
| | Quality of life: physical summary scale | RAND-36: physical summary | 0, 12 months | I: 52.8 (40.1)<br>C: 54.4 (42.8) | I: 47.9 (17.6)<br>C: 50.4 (18.2) | Yes |
| | Quality of life: mental summary | RAND-36: mental summary | 0, 12 months | I: 75.1 (26.2)<br>C: 73.0 (30.5) | I: 74.9 (21.0)<br>C: 72.2 (12.2) | No |
