# Supplementary material for: Blended Interventions to Change Behavior in Patients With Chronic Somatic Disorders: Systematic Review
Source: J Med Internet Res. 2017 Dec 21;19(12):e418. doi: 10.2196/jmir.8108 (PMC5754569; doi:10.2196/jmir.8108)
Supplement: Multimedia Appendix 6 [file jmir_v19i12e418_app6.pdf]

Author, year of publication	Outcome measure	Instrument	Time of measurement (pre- and post-treatment)	Pre-treatment mean (SD)	Post-treatment mean (SD)	Significant effect
**Symptoms and signs**						
Allen, 2013<br>* intensive counseling	Body weight	Weighing scale	0, 6 months	I: 100.3 (16.5)<br>C: 96.0 (17.4)	I: 94.9 (4.0)<br>C: 93.5 (4.1)	No
	BMI	Scale and meter	0, 6 months	I: 34.3 (3.9)<br>C: 34.1 (4.1	I: 32.5 (1.3)<br>C: 33.3 (1.4)	No
	Male waist circumference	Measuring tape	0, 6 months	I: 119.4 (11.6)<br>C: 117.3 (15.5)	I: 112.4 (2.6)<br>C: 114.3 (2.4)	No
	Female waist circumference	Measuring tape	0, 6 months	I: 109.7 (11.4)<br>C: 106.4 (14.5)	I: 104.0 (3.7)<br>C: 103.2 (7.4)	No
Allen, 2013<br>*less intensive counseling	Body weight	Weighing scale	0, 6 months	I: 96.8 (14.8)<br>C:96.0 (17.4)	I: 93.5 (5.9)<br>C: 93.5 (4.1)	No
	BMI	Scale and meter	0, 6 months	I: 33.5 (3.5)<br>C: 34.1 (4.1)	I: 32.4 (2.0)<br>C: 33.3 (1.4)	No
	Male waist circumference	Measuring tape	0, 6 months	I:116.4 (4.6)<br>C: 117.3 (15.5)	I: 109.9 (0.35)<br>C: 114.3 (2.4)	No
	Female waist circumference	Measuring tape	0, 6 months	I: 108.7 (8.4)<br>C: 106.4 (14.5)	I: 105.1 (7.9)<br>C: 103.2 (7.4)	No
De Boer, 2014	Pain-intensity	Visual analogue scale	0, 4 months	I: 6.59 (1.94)<br>C: 5.61 (1.94)	I: 5.19 (2.53)<br>C: 5.49 (2.32)	No
	Extend of fatigue	Visual analogue scale	0, 4 months	I: 6.34 (2.28)<br>C: 6.63 (2.23)	I: 5.91 (2.44)<br>C: 6.88 (2.32)	No
	Mean arterial blood pressure	Blood pressure test	0, 4 months	I: 95.1 (0.8)<br>C: 95.4 (0.8)	I: 94.4 (0.9)<br>C: 94.6 (0.9)	Yes
Nordin, 2016	Pain	Visual Analogue Scale (average of 7 days)	0, 4 months	I: 66.1 (16.7)<br>C: 64.7 (16.2)	I: 59.6 (21.0)<br>C: 54.8 (21.9)	No

**Daily activity related limitations**						
Allen, 2013 * intensive counseling	Self-reported activity	Stanford 7-Day Physical Activity Recall	0, 6 months	I:4.9 (5.7) C:5.0 (5.2)	I: 2.9 (5.4) C: 3.6 (7.1)	No
Allen, 2013 *less intensive counseling	Self-reported activity	Stanford 7-Day Physical Activity Recall	0, 6 months	I: 5.3 (5.4) C:5.0 (5.2)	I:1.7 (5.5) C: 3.6 (7.1)	No
Jasper, 2014	Insomnia	Insomnia Severity Index	0, 10 weeks	I: 12.68 (5.91) C: 12.40 (6.08)	I: 8.70 (5.80) C: 9.03 (6.75)	No
Van der Weegen, 2015	Physical activity	Pam accelerometer	0, post-intervention	I: 39.29 (18.1) C: 47.47 (26.5)	I: 48.16 (23.8) C: 46.28 (30.8)	Yes
**Dealing with the chronic condition: cognitive measures**						
De Boer, 2014	Pain-related catastrophizing	Pain catastrophizing scale	0, 4 months	I: 19.82 (13.9) C: 20.38 (11.38)	I: 11.00 (11.49) C: 16.10 (11.56)	Yes
	Catastrophizing	Pain coping and cognition list	0, 4 months	I: 3.12 (0.72) C: 3.19 (0.89)	I: 2.57 (0.86) C: 3.11 (0.88)	Yes
	Pain coping	Pain coping and cognition list	0, 4 months	I: 3.17 (0.96) C: 3.00 (0.64)	I: 3.72 (0.79) C: 3.14 (0.61)	Yes
	Internal pain management	Pain coping and cognition list	0, 4 months	I: 3.55 (0.67) C: 3.15 (0.98)	I: 4.30 (0.73) C: 3.57 (0.84)	Yes
	External pain management	Pain coping and cognition list	0, 4 months	I: 2.23 (0.86) C: 2.59 (0.94)	I: 1.99 (0.63) C: 2.40 (0.92)	Yes
Jasper, 2014	Acceptance	Tinitis Acceptance Questionnaire	0, 10 weeks	I: 42.07 (11.70) C: 40.26 (11.87)	I: 47.91 (11.70) C: 46.31 (11.96)	No
Nordin, 2016	Self-efficacy pain	Arthritis Self-Efficacy Scale: pain subscale	0, 4 months	I: 45.8 (21.6) C: 49.0 (20.4)	I: 50.0 (23.4) C: 49.3 (21.9)	Yes
	Self-efficacy other symptoms	Arthritis Self-Efficacy Scale: pain subscale	0, 4 months	I: 52.6 (19.2) C: 52.0 (16.7)	I: 58.1 (21.5) C: 56.1 (19.8)	No
	Self-efficacy general	General Self-Efficacy Scale	0, 4 months	I: 2.90 (0.60) C: 2.97 (0.46)	I: 2.88 (0.58) C: 3.06 (0.53)	No

	Coping strategy: diverting attention	Coping Strategy Questionnaire: subscale diverting attention	0, 4 months	I: 2.9 (1.4)<br>C: 2.8 (1.5)	I: 3.2 (1.4)<br>C: 2.9 (1.7)	No
	Coping strategy: reinterpret pain sensation	Coping Strategy Questionnaire: subscale reinterpret pain sensation	0, 4 months	I: 1.8 (1.4)<br>C: 1.7 (1.4)	I: 2.1 (1.3)<br>C: 1.8 (1.4)	No
	Coping strategy: catastrophizing	Coping Strategy Questionnaire: subscale catastrophizing	0, 4 months	I: 3.2 (1.4)<br>C: 2.8 (1.2)	I: 2.8 (1.4)<br>C: 2.8 (1.4)	Yes
	Coping strategy: ignore pain sensations	Coping Strategy Questionnaire: subscale ignore pain sensations	0, 4 months	I: 2.7 (1.2)<br>C: 2.8 (1.2)	I: 2.9 (1.1)<br>C: 2.9 (1.3)	Yes
	Coping strategy: praying or hoping	Coping Strategy Questionnaire: subscale praying or hoping	0, 4 months	I: 2.7 (1.6)<br>C: 2.6 (1.5)	I: 2.8 (1.6)<br>C: 2.5 (1.7)	No
	Coping strategy: self-statements	Coping Strategy Questionnaire: subscale ignore pain sensations	0, 4 months	I: 3.1 (1.1)<br>C:3.1 (1.3)	I: 3.0 (1.2)<br>C: 2.9 (1.3)	No
Van der Weegen, 2015	General self-efficacy	General Self-Efficacy Scale	0, post-intervention	I: 3.2 (0.5)<br>C: 3.2 (0.5)	I: 3.3 (0.4)<br>C: 3.3 (0.5)	No
	Exercise self-efficacy	Exercise Self-Efficacy Scale	0, post-intervention	I: 55.4 (17.0)<br>C: 53.1 (21.3)	I: 59.7 (17.3)<br>C: 59.7 (19.6)	No
**Dealing with the chronic condition: behavioral measures**						
De Boer, 2014	Pain interference with daily activities	Visual analogue scale	0, 4 months	I: 5.89 (2.14)<br>C: 5.93 (2.40)	I: 5.13 (2.52)<br>C: 6.33 (2.21)	No
Nordin, 2016	Coping strategy: Increased	Coping Strategy Questionnaire: subscale praying	0, 4 months	I: 3.3 (1.1)<br>C: 3.1 (1.3)	I: 3.4 (1.0)<br>C: 2.9 (1.3)	No

	behavioral activities	or hoping				
**Emotional outcomes**						
Jasper, 2014	Tinnitus associated distress	Mini-Tinnitus Questionnaire	0, 10 weeks	I: 12.20 (4.58)<br>C: 14.19 (4.51)	I: 7.44 (5.30)<br>C: 8.09 (4.93)	No
	Tinnitus associated distress	Tinnitus Handicap Inventory	0, 10 weeks	I: 40.34 (17.64)<br>C: 44.33 (19.17)	I: 26.67 (20.75)<br>C: 27.70 (21.93)	No
	Anxiety	Hospital Anxiety and Depressiona Scale	0, 10 weeks	I: 7.41 (3.56)<br>C: 7.79 (3.73)	I: 5.44 (3.23)<br>C: 5.84 (3.82)	No
	Depression	Hospital Anxiety and Depressiona Scale	0, 10 weeks	I: 5.95 (4.21)<br>C: 6.02 (3.79)	I: 4.41 (3.72)<br>C: 4.41 (3.92)	No
**Quality of life**						
Van der Weegen, 2015	Health related quality of life: Role impairment (physical)	RAND-36 subscale Role impairment (physical)	0, post-intervention	I: 42.5 (11.1)<br>C: 46.1 (9.8)	I: 45.2 (9.5)<br>C: 46.8 (10.0)	No
	Health related quality of life: Role impairment (emotional)	RAND-36 subscale Role impairment (emotional)	0, post-intervention	I: 48.2 (10.3)<br>C: 48.6 (11.7)	I: 48.8 (10.6)<br>C: 51.6 (11.3)	No
